# Software for pre-processing Illumina next-generation sequencing short read sequences

**DOI:** 10.1186/1751-0473-9-8

**Published:** 2014-05-03

**Authors:** Chuming Chen, Sari S Khaleel, Hongzhan Huang, Cathy H Wu

**Affiliations:** 1 Center for Bioinformatics and Computational Biology, University of Delaware, Newark, DE USA; 2 Geisel School of Medicine, Dartmouth College, Hanover, NH USA

**Keywords:** Next-generation sequencing, Illumina, Trimming, *De novo* assembly, Reference-based assembly, Perl

## Abstract

**Background:**

When compared to Sanger sequencing technology, next-generation sequencing (NGS) technologies are hindered by shorter sequence read length, higher base-call error rate, non-uniform coverage, and platform-specific sequencing artifacts. These characteristics lower the quality of their downstream analyses, e.g. *de novo* and reference-based assembly, by introducing sequencing artifacts and errors that may contribute to incorrect interpretation of data. Although many tools have been developed for quality control and pre-processing of NGS data, none of them provide flexible and comprehensive trimming options in conjunction with parallel processing to expedite pre-processing of large NGS datasets.

**Methods:**

We developed *ngsShoRT* (next-generation sequencing Short Reads Trimmer), a flexible and comprehensive open-source software package written in Perl that provides a set of algorithms commonly used for pre-processing NGS short read sequences. We compared the features and performance of *ngsShoRT* with existing tools: *CutAdapt*, *NGS QC Toolkit* and *Trimmomatic*. We also compared the effects of using pre-processed short read sequences generated by different algorithms on *de novo* and reference-based assembly for three different genomes: *Caenorhabditis elegans, Saccharomyces cerevisiae S288c,* and *Escherichia coli O157 H7*.

**Results:**

Several combinations of *ngsShoRT* algorithms were tested on publicly available Illumina GA II, HiSeq 2000, and MiSeq eukaryotic and bacteria genomic short read sequences with the focus on removing sequencing artifacts and low-quality reads and/or bases. Our results show that across three organisms and three sequencing platforms, trimming improved the mean quality scores of trimmed sequences. Using trimmed sequences for *de novo* and reference-based assembly improved assembly quality as well as assembler performance. In general, *ngsShoRT* outperformed comparable trimming tools in terms of trimming speed and improvement of *de novo* and reference-based assembly as measured by assembly contiguity and correctness.

**Conclusions:**

Trimming of short read sequences can improve the quality of *de novo* and reference-based assembly and assembler performance. The parallel processing capability of *ngsShoRT* reduces trimming time and improves the memory efficiency when dealing with large datasets. We recommend combining sequencing artifacts removal, and quality score based read filtering and base trimming as the most consistent method for improving sequence quality and downstream assemblies.

*ngsShoRT* source code, user guide and tutorial are available at http://research.bioinformatics.udel.edu/genomics/ngsShoRT/. *ngsShoRT* can be incorporated as a pre-processing step in genome and transcriptome assembly projects.

## Background

As a cost-effective, high-throughput alternative to classical Sanger sequencing technology, emerging next-generation sequencing technologies have revolutionized biological research. When compared to Sanger sequencing technology, NGS platforms (e.g. 454, Illumina and ABI-SOLiD) [1] have their drawbacks, including shorter sequence read length, higher base-call error rate, non-uniform coverage and platform-specific artifacts [2-4] that can severely affect the downstream data analysis efforts.

One of the most important areas of NGS data analysis is *de novo* genome or transcriptome assembly. *De novo* assembly is essential for studying non-model organisms where a reference genome or transcriptome is not available. A common approach for *de novo* assembly of NGS sequences uses *De Bruijn* Graph (DBG) [5] data structure, which manages the large volume and short read length of NGS data better than classical Overlap-Layout-Consensus assemblers such as TIGR and Phrap [6,7]. In the DBG-based approach, reads are decomposed into K-mers that in turn become the nodes of a DBG. Sequencing errors complicate the DBG because a single mis-called base can result in a new K-mer sequence that will subsequently introduce a new path in the DBG. These incorrect K-mers increase the complexity of the DBG, prolong assembler runtime, increase memory footprint, and ultimately lead to poor quality assembly [8]. Pre-processing NGS reads to remove mis-called bases would be beneficial to DBG assembler performance and the resulting assembly.

Another important area of NGS data analysis is reference-based assembly i.e. mapping or aligning reads to a reference genome or transcriptome. This step is crucial for many NGS applications including RNA-Seq [9], ChIP-Seq [10], and SNP and genomic structural variant detection [11]. The correct mapping of reads to a reference depends heavily on read quality [12,13]. For example, some mapping tools use the base quality scores of a read to determine mismatch locations. Chimeric reads or other sequencing artifacts can introduce gaps in the alignment. Erroneous bases add additional complexity to the correct identification of actual mismatch positions during the mapping process. Therefore, cleaning up raw sequencing reads can improve the accuracy and performance of alignment tools.

We developed *ngsShoRT* (next-generation sequencing Short Reads Trimmer), a flexible and comprehensive open-source software package that implements many commonly used pre-processing algorithms gathered from the sequencing and assembly literature. In addition, we performed systematic assessments of the effects of using pre-processed short read sequences generated by different algorithms on the resulting *de novo* and reference-based assembly of three genomes: *Caenorhabditis elegans, Saccharomyces cerevisiae S288c,* and *Escherichia coli O157 H7*. We also compared the performance of *ngsShoRT* with other existing trimming tools: *CutAdapt*[14], *NGS QC Toolkit*[15] and *Trimmomatic*[16].

## Methods

### Overview

For a typical NGS data analysis pipeline, *ngsShoRT* serves as a module between the raw sequences generated by NGS sequencers and further downstream analyses (Figure S1) [see Additional file 1].

*ngsShoRT* takes Single-Read (SR), Paired-End (PE), and Mate-Pair (MP) FastQ or Illumina’s native QSEQ format sequence files as input (compressed files are supported also) and runs them through a set of independent pre-processing algorithms including adapter/primer sequence removal, homopolymer sequence removal, Illumina QSEQ specific methods, reads with “N” bases removal/splitting, quality score based trimming, and 5' or 3'-end bases trimming. Outputs include a set of SR or PE/MP reads in FastQ format and a detailed summary statistics report. Using *ngsShoRT* to pre-process short read sequences is usually an iterative process: raw reads are trimmed by one of the *ngsShoRT* methods and the output can be used as an input to another *ngsShoRT* method. The end product is a trimmed data set ready for incorporation into various downstream assembly and data analysis pipelines.

### Design principles

#### Common pre-processing issues

There are several types of potential errors in NGS reads: un-called “N” bases, sequencing artifacts (usually platform specific PCR primers, linkers and adaptors) and low quality bases. Errors are more likely to occur at the 3′-ends of a read from Illumina sequencing technology [2].

Many *de novo* genome assembly projects [15-21] included pre-processing steps for removing reads with un-called “N” bases, and detecting and removing of adaptor sequences using exact string matching algorithms to search for user-specified adaptor sequences. However, exact string matching may fail to detect all adapter sequences because of sequencing errors. A customizable approximate matching algorithm is more desirable in this case.

Another issue of NGS read pre-processing is dealing with PE or MP reads that are important for repeat resolution and scaffolding used by the DBG assemblers. The PE or MP reads are usually partitioned into two separate files with forward direction reads in the first file and reverse direction mate reads in the second file. The reads are listed *in the same order* in both files, an assumption critical to the assembly process. Surprisingly, most existing pre-processing tools do not process PE or MP read files as a single unit (Table 1). Instead, they treat each file separately, which can result in removing some reads from the first file, but not their corresponding mates in the second file, resulting in a loss of “*same order”* read pairing. The pairing is also usually lost if either trimmed read is shorter than the K-mer length parameter used by the DBG assembler [22]. A preferred approach would be to divert “widowed” mates of PE or MP reads (reads whose mates were removed during trimming) to a separate single read file, while maintaining read ordering in the trimmed PE or MP read files. In addition, because maintaining the post-trimming read length to be ≥ K is critical for DBG assemblers (e.g., Velvet), trimming algorithms need to be adjusted to ensure that they do not trim reads to be of lengths shorter than K.

**Table 1:** **Comparison of** ***ngsShoRT*** **with other publically available pre-processing tools**

**Tool**	**Programming language**	**Targeted NGS platform**	**Input format**	**PE reads handling**	**Parallel processing**	**NGS artifacts handling**	**Quality score-based trimming**	**Output format**	**Summary report**
**ngsShoRT (2.1)**	Perl	454, Illumina	FastQ, Illumina QSEQ	Yes	Yes	Yes	Yes: 3'-end, quality window and filter out low quality reads	FastQ	Yes
**NGS QC toolkit (v.2.3.2) **[15]	Perl	454, Illumina^1^	FastQ, FastA (+ .qual)	Yes	Yes	Yes	Yes: filter out low quality reads	FastA (+.qual), FastQ	Yes
**FASTX toolkit (v. 0.0.13.2) **[26]	C/C++	Non-specific	FastQ^2^, FastA (not .qual)	No	No	No	Yes: filter out low quality reads	FastA, FastQ	No
**SeqTrim **[25]	Perl	Non-specific^3^	FastA (+ .qual), Phred	No	No	No	Yes: filter out low quality reads	FastA (+.qual)	Yes
**CutAdapt (v.1.3) **[14]	Python^4^	454, Illumina, SOLID^5^	FastQ, SOLID’s cs.FastA + cs.FastA.qual	No	No	No	Yes: filter out low quality reads	FastQ, SOLID’s cs.FastA + cs.FastA.qual	No
**Btrim **[27]	C++^6^	Illumina	FastQ^6^	No	No	No	Yes: quality window	FastQ	No
**SolexaQA (v.2.2) **[8]	Perl	Illumina	FastQ	Yes	No	No^7^	Yes: quality window and filter out low quality reads	FastQ	Yes
**Sickle **[28]	C/C++^8^	Illumina	FastQ	Yes	No	No	Yes: quality window	FastQ	Yes
**Scythe **[24]	C/C++^8^	Illumina	FastQ	No	No	Yes, but only 3’	No	FastQ	Yes
**Trimmomatic (v.0.32) **[16]	Java	Illumina	FastQ	Yes	Yes	Yes	Yes: quality window and filter out low quality reads	FastQ	Yes

^1^NGS QC’s IlluQC only works for FastQ file, and 454QC only works for FastA (+.qual) file [15].

^2^FASTX toolkit does not accept multi-line FastQ file and requires reformatting to one-line FastQ file using provided tools [26].

^3^While SeqTrim isn’t platform-specific, it can only take FastA file (with/without .qual and chromatogram) [25].

^4^Most of CutAdapt is in python, but the alignment algorithm was written in C for speedup [14].

^5^CutAdapt was designed with RNA-Seq technology in mind [14].

^6^Btrim’s C++ implementation is designed for single reads. The tool website offers an un-optimized Perl script that organizes separately trimmed paired-end files [27].

^7^SolexaQA does not provide primer/adapter trimming [8].

^8^Scythe and Sickle require Zlib (http://www.zlib.net/) [24,28].

Earlier versions of Illumina sequencer (prior to Casava 1.8, released in 2011) produced sequences in QSEQ format instead of FastQ format [23]. A unique feature of QSEQ format read is its “Failed_Chastity” filter flag, which indicates it is a low quality read. Additionally, QSEQ format used an ASCII-to-Phred quality score mapping where ASCII character #64 corresponds to a Phred quality score of zero. In this mapping, a “B” character is a special indicator for “unknown quality score”. Both the “Failed_Chastity” and “B” character flags are lost when reads in QSEQ format are converted to more popular FastQ format, which uses a “Sanger-based” ASCII-to-Phred mapping where ASCII character #32 corresponds to a Phred quality score of zero. It would be useful for the trimming tools that target the Illumina platform to be able to process Illumina’s native QSEQ format read for datasets generated prior to Casava 1.8.

Finally, given the large volume of data generated by NGS sequencers, another compelling feature for a NGS short read pre-processing software would be its scalability and the capability of parallel processing to reduce computational time.

#### Review of currently available trimming tools

We reviewed nine publicly available NGS pre-processing tools [8,14-16,24-28] for: targeted NGS platforms, input/output formats supported, ability to handle PE or MP reads generically, scalability, sequencing artifacts handling, types of quality score based trimming methods supported, and finally, output of trimming and QC statistics summary report (Table 1). Many tools trim a number of bases from the 3'-end of a read because in general, low quality bases occur more frequently at 3'-end of a read [8,20,25]. A more refined approach involves a sliding window algorithm that tries to extract a substring of read bases where the first and last base’s quality scores exceed a specified cutoff [8,25]. The quality score based window extraction approach is somewhat arbitrary when it comes to determining the quality score cutoff and the window size. For example, specifying a window size shorter than the K-mer length used by the DBG assembler will result in skipping many of the trimmed reads altogether [22]. A simpler approach is to extract “high quality reads”, i.e. reads with a percentage of high quality bases (bases whose quality scores exceed a specified cutoff) that satisfies a user-specified percentage cutoff [15,20,25,26]. The problem with this approach is that it may filter out reads that could have been salvaged by trimming fewer low quality bases from their 3'-ends.

To the best of our knowledge, the only freely available tools that handle PE or MP reads generically are Btrim [27] (in the secondary step following separate paired-end reads trimming), SolexaQA (v.2.2) [8], Sickle [28], Trimmomatic [16], and NGS QC Toolkit (v.2.3) [15]. Of these tools, only Trimmomatic supports parallel-processing on the original SE or PE input files, while NGS QC Toolkit supports “parallel” trimming of separate input files by using one thread for each file.

### Algorithms and implementation

*ngsShoRT* provides 12 algorithms/methods in 5 categories summarized in Table 2 and described in detail below.

**Table 2:** **Short read sequence pre-processing algorithms in** ***ngsShoRT***

**Category**	**Algorithm/Method**	**Description**
Sequencing Artifacts Removal	5adpt	Detects (using exact or approximate matching) sequencing artifacts listed in an input file and removes them.
	rmHP	Removes homopolymer sequences.
QSEQ Specific Methods	qseq0	Removes QSEQ reads with “Failed_Chastity” filter flags.
	qseqB	Removes reads with more than certain number of "B"-scored bases.
Reads with “N” Bases Removal/Splitting	nperc	Filters out reads with un-called “N” bases exceeding a percentage cutoff.
	ncutoff	Filters out reads with un-called “N” bases exceeding a number cutoff.
	nsplit	Searches and removes “N” bases, then splits the read around the removed “N” bases into two smaller daughter reads.
Quality Score Based Trimming	LQR	Removes “low quality” reads using quality score cutoff or percent cutoff.
	Mott	Quality-window extraction (trim both the 5'- and 3'-ends of a read).
	TERA	Trims low quality-score bases from the 3'-ends of reads based on their running average quality scores.
5'/3'-end Bases Trimming	3end	Trims bases from 3'-end of a read.
	5end	Trims bases from 5'-end of a read.

#### Sequencing artifacts removal

5adpt [*mp*, *list*, *approx_match_modifiers*, *search_depth*, *action*] detects (at a match percentage *mp* and up to a depth of *search_depth*) 5'-adaptor/primer sequences loaded from a *list* (which can be built-in Illumina primer library, and/or user-provided sequences) and removes them from a read. 5adpt allows users to do approximate matching using the Levenshtein edit distance implementation in CPAN’s *String::Approx* module [29]. This module allows approximate matching using a simple percentage cutoff or detailed modifiers (number of allowed insertions, substitutions, and deletions). This feature, accessible through the *approx_match_modifiers* option, allows 5adpt to be modified to fit platform specific features and error profiles. For example, one would expect more InDels over substitutions in 454 reads, and the opposite in Illumina reads [30]. After an adapter/primer/linker sequence (or fragment) is matched and trimmed from a read, the *action* option allows users to specify how to handle this read. A read can be removed completely (*action* = kill-read, kr) or trimmed to the base 5' to the detected artifact string (*action* = kill-after, ka).

rmHP [*h*, *b*] searches for homopolymer sequences whose lengths are ≥ *h* in the reads and are composed of bases listed in *b* (normally A, C, G, and T). If detected, the homopolymer sequence and all bases 3' of it are removed from the read.

#### QSEQ specific methods

qseq0 and qseqB [*n*, *mode*, *action*] are methods we developed specifically for reads in Illumina QSEQ format, the default output format for Illumina sequencer prior to Casava 1.8 [23]. qseq0 removes a QSEQ read that does not pass the “Failed_Chastity” filter, which was shown to greatly improve assembly contiguity and correctness [31]. qseqB trims a read with more than *n* "B"-scored bases. Because a “B” score means “unknown quality score”, these bases are usually trimmed along with all the bases 3' to them [18,32]. Unfortunately, conversion of QSEQ format to popular FastQ format results in losing the “Failed_Chastity” filter flag information. In addition, conversion usually includes changing the ASCII score mapping from Illumina-based to Sanger-based mapping, which changes the “B” quality score to a different ASCII character. Therefore, unlike qseq0, qseqB still can be used with Illumina reads in FastQ format if their ASCII-to-Phred score mapping could be switched from Sanger back to Illumina using the switch_score method (see below). At *mode* = “local”, qseqB [*n*] will remove a read with ≥ *n* "B"-scored bases. qseqB [*n*] will search for a string of consecutive "B"-scored bases no shorter than *n*. If such a string is detected, the read can be removed completely (*action* = kill-read, kr) or trimmed to the base 5' to the detected "B"-scored string (*action* = kill-after, ka). A QSEQ limited implementation of qseqB with *mode* = local and *action* = ka was used by Garcia et al. [32].

switch_score is not a trimming method, but it allows switching the ASCII-to-Phred mapping of a base-call quality score between Illumina’s legacy ASCII-64-based mapping and Sanger’s ASCII-32-based mapping. This restores the original Illumina quality scores for the read bases in FastQ format downloaded from NCBI’s Sequence Read Archive, including the aforementioned "B"-scored bases that then can be trimmed using qseqB method.

#### Reads with “N” bases removal/splitting

nperc [*p*] and ncutoff [*n*] filter out a read with un-called “N” bases where the percentage or number of “N” bases is ≥ *p* or ≥ *n*, respectively. ncutoff [*n* =1] is equivalent to the commonly used pre-processing step of filtering out reads that contain “N” bases.

nsplit [*l*] detects a string of un-called “N” bases whose length is ≥ *l*, removes them from a read and splits the read around the detected “N” bases into two smaller daughter reads. We developed this method to remove “N” bases from a read without having to filter out the entire read and lose its information.

nperc, ncutoff and nsplit are important for removing “N” bases from a read because they are usually associated with low quality score bases, and DBG assemblers either discard reads with such bases [33] or simply convert them to an arbitrarily chosen nucleotide such as “A” [22].

#### Quality score based trimming

LQR [*lqs*, *p*] trims a “low quality” read using a low quality score (*lqs*) cutoff for individual bases, and a percentage cutoff (*p*) to limit the number of such bases in a read. LQR filters out a read with over *p*% of bases whose quality score is under *lqs*. It is similar to the algorithms used in other pre-processing tools [16,26,34].

Mott [*ml*] is a quality window extraction algorithm that trims from both the 5'- and 3'-ends of a read. Starting at the 3'-end of a read, it counts the running sum of (*ml* - P_*error*_) values, RSMLP, for each base (P_*error*_ of a base = 10^-qulityscore/10^) in the read, and extracts the string from the first base with RSMLP > 0 to the base with the highest RSMLP. Mott algorithm was adapted from the CLC Bio Genomics Workbench [34]. Mott is also similar to the “QRL” algorithm used by DiGuistini et al. [18].

TERA [*avg*] is an algorithm we developed as an alternative to the 3end method (see below). Unlike 3end, TERA trims the 3'-end of a read differently depending on its bases’ quality scores. Starting at the 3'-end, the running average quality score (RAQS) of each base is calculated until it exceeds a cutoff, *avg*, at the base X. All bases 3' to X are then discarded. A good read with high quality (above *avg*) bases at its 3'-end would have fewer bases trimmed by TERA, while a low quality read might have more bases trimmed.

#### 5'/3'-end bases trimming

3end [*x*] trims *x* bases from the 3'-end of a read, 5end [y] trims *y* bases from the 5'-end of a read.

#### Adjustment for DBG assembly

To avoid trimming a read to be of length shorter than the K-mer size used by DBG assemblers, *ngsShoRT* enforces a global minimum read length limit, *min_rl*, on TERA, 3end, 5end, and Mott methods to stop trimming once a read’s remaining length reaches *min_rl*. For example, if the highest K-mer length used for assembly is 41 bps, the user should set *min_rl* to be that value or larger. Another special case to handle is “widowed” mates. If a paired-end read had only one read of the pair filtered, *ngsShoRT* saves this “widowed” read in a separate single read file that can be co-assembled with rest of PE or MP reads. This approach was suggested by Daniel Zerbino at EMBL-EBI (personal communication) and has been used by other genome assembly projects [18,35].

#### Implementation

*ngsShoRT* supports parallel processing by using multi-threading to deal with large volume of data and reduce trimming time. Another unique feature of *ngsShoRT* is its ability to handle PE or MP reads generically using paired-end specific modules.

*ngsShoRT* is implemented using object-oriented Perl 5.6 where the main object is a READ object. Every time *ngsShoRT* parses a read from the input read file (QSEQ or FastQ format), a READ object is created to hold its attributes (header, sequence bases, quality scores of the corresponding bases, and “Failed_Chastity” filter flag in the case of QSEQ format read). Pre-processing methods act on the READ object’s attributes and are independent of the read’s original file format, which makes it easy to implement additional pre-processing algorithms or have different output formats. Parallel processing is performed using Perl’s built-in threads module, where each thread processes a separate set of reads from the input file with the processed reads merged in the final step.

### Evaluation

We compared the performance of *ngsShoRT* with three other tools: *CutAdapt*, *NGS QC Toolkit* and *Trimmomatic.* Since these tools implement different processing algorithms, we compared only the algorithms that are similar to the ones in *ngsShoRT* in terms of running speed and RAM usage. We compared the effects of using pre-processed reads generated by these different algorithms on the *de novo* and reference-based assembly of three different genomes: *Caenorhabditis elegans, Saccharomyces cerevisiae S288c* and *Escherichia coli O157 H7*, and evaluated assembly quality and assembler performance. Evaluation workflow is shown in Figure S2 [see Additional file 1].

#### Data source and experimental settings

The short read sequence data of *Caenorhabditis elegans, Saccharomyces cerevisiae S288c* and *Escherichia coli O157 H7* genomes downloaded from NCBI Sequence Read Archive were used for the evaluation. The details of raw sequence data are shown in Table 3. The goal of our evaluation experiments is to take raw short read sequences for different organisms generated by multiple NGS platforms, process them using different pre-processing algorithms, then do *de novo* and reference-based assemblies. Because all three selected organisms have reference genomes, we can then evaluate the quality of our *de novo* and reference-based assemblies against the reference genomes. The experiments were conducted on X86_64 Fedora 17 server with 256G RAM and 32 Intel(R) Xeon(R) CPU/Core X7550 @ 2.00GHz.

**Table 3:** The descriptions of raw short-read sequences used in the evaluation experiments

**DataSet**	***Caenorhabditis elegans***	***Saccharomyces cerevisiae S288c***	***Escherichia coli O157 H7***
**Taxonomy ID**	6239	559292	83334
**Reference Genome size (bp)**	100.3 M	12.2 M	5.5 M
**#Chromosomes**	7*	17*	1
**SRA run**	SRR065390	SRR449310	SRR957847
**Platform**	Illumina Genome Analyzer II	Illumina HiSeq 2000	Illumina MiSeq
**Strategy**	WGS	WGS	WGS
**Source**	Genomic	Genomic	Genomic
**Layout**	Paired	Paired	Paired
**Read length**	100	76	150
**Nominal length**	356	230	350
**Total sequences (paired)**	33,808,546	1,898,259	2,241,778
**Total bases (paired)**	6,761,709,200	288,535,368	672,533,400
**Mean Phred quality score**	29.49	34.17	33.12
**Low Phred quality score (<=10)**	1,902,576 (2.81%)	167,669 (4.42%)	76,598 (1.71%)
**Coverage**	67.4x	23.7x	122.3x
**GC content (%)**	35	39	50

*The mitochondrial chromosome is included.

#### Comparison of ngsShoRT algorithms

We applied 5 basic *ngsShoRT* pre-processing algorithms (5adpt, TERA, 3end, LQR, Mott) and some of their combinations (LQR-5adpt, LQR-5adpt-TERA, LQR-5adpt-Mott, 3end-TERA, 3end-nX etc.) to the raw sequences of three organisms to generate pre-processed data sets. We collected runtime and peak RAM usage used to generate each pre-processed sequence data set and computed the mean quality score and sequence/read counts of each experimental data set.

We evaluated the multi-threading performance of *ngsShoRT* by running the Mott algorithm (the slowest one of all the algorithms provided by *ngsShoRT*) on *Caenorhabditis elegans* GAIIx raw sequences with 1, 8, 16 or 32 threads. We recorded total runtime, maximum thread runtime, final merging runtime, and peak RAM usage.

#### Comparison with other tools

We compared *ngsShoRT* with other tools in three categories of algorithms: 1) adapter trimming, 2) 3'-end trimming, and 3) quality score based trimming. For the adapter trimming, we compared *ngsShoRT* with *CutAdapt*, *NGS QC Toolkit* and *Trimmomatic*. For 3'-end trimming, we compared *ngsShoRT* with *NGS QC Toolkit*. For quality score based trimming, we compared *ngsShoRT* with *NGS QC Toolkit* and *Trimmomatic*. We also collected runtime and peak RAM usage used to generate each pre-processed sequence set, as well as its corresponding mean quality score and sequence/read counts.

#### De novo assembly

We performed DBG-based *de novo* assembly of raw and pre-processed sequences of three organisms using the popular Velvet assembler [22] (v1.2.10, with OPENMP enabled). We ran Velvet using the VelvetOptimiser [36] (v2.2.5), which automatically optimizes the three key parameters of velvet: K-mer_length, expected_coverage, and coverage_cutoff. As suggested in GAGE [37], we excluded “chaff” contigs (single contig less than 200 bps in length) and computed “E-size”, which is the expected size of the contig containing a given random location in the reference genome.

We assessed the correctness of assemblies by aligning the assembled contigs to corresponding reference genome using the methods of GAGE [37]. We used the *nucmer* aligner from MUMmer v3.23 [38] to construct local pairwise alignments between reference genome sequences and assembled contigs with the options “-maxmatch -l 30 -banded -D 5”. Alignments with less than 95% identity or more than 95% overlap with another alignment were discarded using *delta-filter*. The statistics of remaining alignments were computed by *dnadiff*[39] using default parameters.

#### Reference-based assembly

We completed reference-based assembly of raw and pre-processed sequences of three organisms using BWA-MEM algorithm of BWA v0.7.5a [12]. The alignment and accuracy statistics were computed by QualiMap v0.7.1 [40].

## Results

### Performance of different ngsShoRT algorithms

A summary of the trimmed data sets generated by different *ngsShoRT* algorithms is shown in Figure S3 [see Additional file 1] and Tables SA1-1, SA1-2, and SA1-3 [see Additional file 2]. In terms of the total number of bases removed from the raw sequences, 3end method removed the most and 5adpt method removed the least. The mean quality scores of pre-processed sequences are all increased, with the highest read quality resulting from TERA and its combination with other methods. LQR and its combination with other methods removed all the low quality score (<=10) bases. 5adpt and Mott methods as well as their combination with other methods took longer time to run and required more RAM.

The summary of comparing *ngsShoRT* algorithms to other tools with similar algorithms is shown in Figure S4 [see Additional file 1] and Tables SA5-1, SA5-2, and SA5-3 [see Additional file 3]. In the three categories of algorithms we compared, *ngsShoRT* algorithms removed more low quality bases, improved the overall quality scores of the trimmed data, and required less time and RAM to run.

The results of multi-threading performance of *ngsShoRT* are listed in Table 4. As the number of threads increases, the total runtime of *ngsShoRT* job in terms of running the relatively time-consuming Mott algorithm on *Caenorhabditis elegans* data set decreases and the peak RAM usage increases linearly. In contrast, the runtime of the final merging step remains relatively constant regardless of the number of threads used. This indicates that the most computationally intensive work is trimming the reads by each thread, not merging the outputs from threads.

**Table 4:** **Multi-threading performance of** ***ngsShoRT***

**# threads**	**Total runtime (mins.)**	**Peak RAM usage (MB)**	**Max thread runtime (mins.)**	**Merging runtime (mins.)**
1	385.567	9.86	NA	NA
8	58.733	26.49	47.950	10.650
16	36.850	44.93	26.433	10.283
32	25	81.96	14.333	10.550

### Effects on de novo and reference-based genome assembly

#### 1) de novo genome assembly

The summary of *de novo* genome assemblies of raw and trimmed data generated by *ngsShoRT* algorithms is shown in Figure S5 [see Additional file 1] and Tables SA2-1, SA2-2, and SA2-3 [see Additional file 2]. Comparing to raw sequence assemblies, trimmed sequence assemblies had better assembly continuity in terms of total and max contig length, N50, and E-size, with the exception of the assemblies of pre-processed *Escherichia coli O157 H7* sequences by Mott and LQR methods. In addition, the assemblies of pre-processed sequences ran faster and required less RAM than the assemblies using raw data. Using pre-processed data improved the accuracy of assemblies as shown in Figure S6 [see Additional file 1] and Tables SA3-1, SA3-2, and SA3-3 [see Additional file 2]. More reference genome is covered by the assembled contigs using pre-processed data, and more contigs can be aligned to the reference with the exception of contigs assembled from datasets generated by 3end and Mott methods. This may be explained by the fact that these methods had the highest percentage of trimmed bases and shorter reads relative to other trimming methods, suggesting lower genome coverage. Overall, the assembled contigs using pre-processed data had fewer SNPs, particularly for the *Sacchariomyces cerevisiae S288c* data sets. The assemblies of pre-processed data by 5adpt, TERA, Mott methods and their combination with LQR method had more SNPs.

Assemblies using raw and pre-processed sequences by *ngsShoRT* and other tools are compared in Figure S7 [see Additional file 1] and Tables SA6-1, SA6-2, and SA6-3 [see Additional file 3]. For adapter trimming algorithms, *ngsShoRT* 5adpt method was the best in terms of max contig length, N50, and E-size, but *CutAdapt* performed best for total contig length. *NGS QC Toolkit* used the least amount of RAM. For the 3'-end trimming algorithms, *ngsShoRT* 3end method outperformed *NGS QC Toolkit* in total contig length, Velvet runtime, and peak RAM usage. *NGS QC Toolkit* outperformed *ngsShoRT* 3end method for max contig length, N50, and E-size. Among quality score based trimming algorithms, *ngsShoRT* quality score based methods outperformed others for Velvet runtime and peak RAM usage. As shown in Figure S8 [see Additional file 1] and Tables SA7-1, SA7-2, and SA7-3 [see Additional file 3], assemblies of 5adpt-trimmed datasets had more contigs aligned to the reference genomes than assemblies from other adapter trimming tools. Overall, assemblies using *NGS QC Toolkit* trimmed reads had fewer SNPs. In the 3'-end trimming algorithms, *ngsShoRT* 3end method performed better in terms of reference genome coverage and number of SNPs in the assembled contigs. In the category of quality score based trimming algorithms, *ngsShoRT* TERA method was the best in reference genome coverage and percent of contigs aligned. *ngsShoRT* Mott method had the smallest number of SNPs in the assembled contigs.

#### 2) Reference-based genome assembly

A comparison of mapping raw sequences and *ngsShoRT* processed sequences to the reference genome using the short-read aligner BWA is shown in Figure S9 [see Additional file 1] and Tables SA4-1, SA4-2, and SA4-3 [see Additional file 2]. Overall, pre-processed sequences had more reads mapped to the reference genome with the exception of data sets generated by TERA and Mott methods. In addition, *ngsShoRT* processed sequences had fewer duplicated reads, clipped reads (reads not completely aligned to the reference from the beginning to the end), and InDel containing reads when mapped to the reference genome. The mapping of pre-processed reads to the reference ran faster than mapping of raw reads.

In the comparison of adapter trimming algorithms of different trimming tools, as shown in Figure S10 [see Additional file 1] and Tables SA8-1, SA8-2, and SA8-3 [see Additional file 3], *NGS QC Toolkit* adapter trimming method had the highest number of pre-processed reads with fewer mismatches and InDels in the mapped reads, as well as shorter mapping time. For the 3'-end trimming algorithms, *ngsShoRT* and *NGS QC Toolkit* 3'-end trimming methods achieved a similar percentage of mapping rate, but *ngsShoRT* trimmed reads had fewer mismatches and InDels, and mapped slower than reads trimmed with *NGS QC Toolkit* 3'-end trimming algorithm. Of the quality score based algorithms, *ngsShoRT* LQR method achieved the highest mapping rate. Datasets pre-processed by *ngsShoRT* TERA and Mott methods had fewer mismatches and InDels within the reads mapped to the reference. *Sacchariomyces cerevisiae S288c* and *Escherichia coli O157 H7* data sets pre-processed by *NGS QC Toolkit*’s quality score based trimming method had the shortest mapping times.

## Discussion

Not surprisingly, removing Illumina sequencing artifacts using 5adpt method improved all assembly measures for all tested tools and datasets. The amount of improvement varied depending on the artifact removing algorithms used. Therefore, to compare a tool’s performance, we used a common library containing known Illumina artifacts for all tested tools to avoid bias against tools that lack built-in artifact libraries. In general, *ngsShoRT’s* 5adpt method outperformed other tools’ adapter trimming algorithms in terms of trimmed dataset assembly contiguity (N50, max contig length, and E-size).

While sequencing artifacts removing algorithms use similar string matching algorithms, quality score based trimming can be implemented using algorithms that emphasize different aspects of a read’s quality scores. Generally speaking, these can be classified into read filtering methods (RF), such as LQR, which removes the entire read based on having a higher percentage of low quality or N bases than a given cutoff and base quality trimming (BQT) methods, such as qCtuoff, qualWindow, qseqB, Mott, and TERA, which attempt to trim low quality bases from one or both ends of a read to produce a higher quality subsequence. As expected, RF methods successfully removed all low quality reads from trimmed datasets while BQT methods did not. In contrast, the mean read quality score was generally higher in BQT trimmed datasets since a “high quality” read filtered out by RF methods may still have low quality bases that can be trimmed by BQT methods, thus improving the overall mean quality score of a read and reducing the number of low quality and potentially erroneous reads. Consequently, *de novo* assembly of BQT trimmed datasets had generally higher N50, max contig length, and percentage of aligned contigs when compared to RF trimmed datasets.

The above results are interesting because most of the assembly projects we examined preferred RF methods, i.e., filtering out all low quality reads, over using BQT methods, i.e., attempting to salvage more reads by trimming their low quality bases. As discussed above, this approach can lead to useful base information being discarded from the “low quality reads” by removing them altogether instead of retaining their higher quality regions. In addition, reads with high average quality score that pass low quality filtering can still contain a small but relevant set of low quality bases that may adversely affect assembly.

Another popular but questionable trimming approach [17,32] is the use of 3'-end trimming to improve assembly contiguity by removing an arbitrary number of bases from the 3'-end of a read. This is based on the assumption that the base quality score, especially in NGS, decreases towards 3'-end of a read and that the decrease in base quality score is uniform for all reads, i.e., that the majority of low-quality bases fall within the arbitrary range of bases removed by 3'-end trimming methods. In our experiments, we compared the trimming of 10 bases using *ngsShoRT* and *NGS QC Toolkit*’s 3'-end trimming methods to the aforementioned BQT methods, set a low quality cutoff of 2, which on average trimmed less than 10 bases per read. BQT methods outperformed 3'-end trimming methods in almost all of our assembly evaluation measures, suggesting that they managed to remove fewer low quality bases overall relative to simple 3'-end trimming.

The performance comparison of multiple *ngsShoRT* trimming algorithms to their counterparts in three other tools: *CutAdapt*, *NGS QC Toolkit*, and *Trimmomatic* showed that *ngsShoRT* overall outperformed them in terms of running speed, quality of trimmed reads, DBG-based *de novo* assembly, and reference-based assembly.

Finally, we tried to determine the best combination of *ngsShoRT* pre-processing algorithms using only methods that considerably improved our evaluation measures in earlier stages of the experiments (ncutoff and nsplit were excluded). Combinations include sequencing artifacts removal using 5adpt in combination with one or more quality score based trimming methods. The compared combinations are shown in Figures S3, S5, S6, and S9 [see Additional file 1]. Interestingly, while adding quality score based trimming to artifacts trimming improved mean read quality scores of trimmed sequences, it did not always improve (and sometime even lowered) N50 and max contig length in *de novo* assembly when compared with artifacts removal alone in the three tested datasets. LQR_5adpt_TERA proved to be the most consistent combination in terms of improving mean read quality, as well as DBG-based *de novo* assembly, and reference-based assembly.

## Conclusions

This paper presents *ngsShoRT*, a flexible and comprehensive open-source software package that implements novel algorithms as well as several short read pre-processing algorithms/methods adapted from literature to pre-process Single-Read and Paired-End/Mate-Pair NGS short reads in FastQ or raw Illumina QSEQ formats. Several combinations of *ngsShoRT* algorithms/methods were tested on publicly available Illumina GA II, HiSeq 2000, MiSeq eukaryotic and bacteria genomic data. In general, contiguity and correctness of the experimental *de novo* assemblies and assembler performance were improved. *ngsShoRT* can be incorporated as a pre-processing step for genome and transcriptome sequencing pipelines to clean up NGS data for downstream data analyses.

We reviewed several commonly used trimming tools and compared the performance of *ngsShoRT* to three of these tools in trimming and assembly (*de novo* and reference-based) of three different genomic datasets generated by three different sequencing platforms. *ngsShoRT* outperformed the compared tools in trimmed read quality as well as DBG-based *de novo* assembly and reference-based assembly.

Finally, based on our trimming evaluation experiments, we recommend combining sequencing artifacts removal, and quality score based read filtering and base trimming as the most consistent method for improving downstream assembly.

## Competing interests

The authors declare that they have no competing interests.

## Authors' contributions

CC, SSK and HH designed the study. SSK developed the software. CC and SSK carried out the evaluation. CHW coordinated the study. CC, SSK, HH and CHW drafted the manuscript. All authors approved the final manuscript.

## Supplementary Material

Additional file 1: Figure S1 Workflow and functional components of *ngsShoRT* software. **Figure S2.** Workflow of DBG-based *de novo* assembly and reference-based assembly evaluation experiments. **Figure S3.** Summary of trimmed data generated by *ngsShoRT* algorithms. **Figure S4.** Summary of trimmed data generated by *ngsShoRT* algorithms and other tools. **Figure S5.** Summary of *de novo* genome assemblies of raw and trimmed data generated by *ngsShoRT* algorithms. **Figure S6.** Correctness of *de novo* genome assemblies of raw and trimmed data generated by *ngsShoRT* algorithms. **Figure S7.** Summary of *de novo* genome assemblies of raw and trimmed data generated by *ngsShoRT* algorithms and other tools. **Figure S8.** Correctness of *de novo* assemblies of raw and trimmed data generated by *ngsShoRT* algorithms and other tools. **Figure S9.** Summary of reference-based assemblies of raw and trimmed data generated by *ngsShoRT* algorithms. **Figure S10.** Summary of reference-based assemblies of raw and trimmed data generated by *ngsShoRT* algorithms and other tools. Click here for file

Additional file 2: Table SA1-1 Summary of *Caenorhabditis elegans* trimmed data generated by *ngsShoRT* algorithms. **Table SA1-2.** Summary of *Saccharomyces cerevisiae S288c* trimmed data generated by *ngsShoRT* algorithms. **Table SA1-3.** Summary of *Escherichia coli O157 H7* trimmed data generated by *ngsShoRT* algorithms. **Table SA2-1.** Summary of *Caenorhabditis elegans de novo* genome assemblies of raw and trimmed data generated by *ngsShoRT* algorithms. **Table SA2-2.** Summary of *Saccharomyces cerevisiae S288c de novo* genome assemblies of raw and trimmed data generated by *ngsShoRT* algorithms. **Table SA2-3.** Summary of *Escherichia coli O157 H7 de novo* genome assemblies of raw and trimmed data generated by *ngsShoRT* algorithms. **Table SA3-1.** Correctness of *Caenorhabditis elegans de novo* genome assemblies of raw and trimmed data generated by *ngsShoRT* algorithms. **Table SA3-2.** Correctness of *Saccharomyces cerevisiae S288c de novo* genome assemblies of raw and trimmed data generated by *ngsShoRT* algorithms. **Table SA3-3.** Correctness of *Escherichia coli O157 H7 de novo* genome assemblies of raw and trimmed data generated by *ngsShoRT* algorithms. **Table SA4-1.** Summary of *Caenorhabditis elegans* reference-based assemblies of raw and trimmed data generated by *ngsShoRT* algorithms. **Table SA4-2.** Summary of *Saccharomyces cerevisiae S288c* reference-based assemblies of raw and trimmed data generated by *ngsShoRT* algorithms. **Table SA4-3.** Summary of *Escherichia coli O157 H7* reference-based assemblies of raw and trimmed data generated by *ngsShoRT* algorithms. Click here for file

Additional file 3: Table SA5-1 Summary of *Caenorhabditis elegans* trimmed data generated by *ngsShoRT* algorithms and other tools. **Table SA5-2.** Summary of *Saccharomyces cerevisiae S288c* trimmed data generated by *ngsShoRT* algorithms and other tools. **Table SA5-3.** Summary of *Escherichia coli O157 H7* trimmed data generated by *ngsShoRT* algorithms and other tools. **Table SA6-1.** Summary of *Caenorhabditis elegans de novo* genome assemblies of raw and trimmed data generated by *ngsShoRT* algorithms and other tools. **Table SA6-2**. Summary of *Saccharomyces cerevisiae S288c de novo* genome assemblies of raw and trimmed data generated by *ngsShoRT* algorithms and other tools. **Table SA6-3.** Summary of *Escherichia coli O157 H7 de novo* genome assemblies of raw and trimmed data generated by *ngsShoRT* algorithms and other tools. **Table SA7-1**. Correctness of *Caenorhabditis elegans de novo* genome assemblies of raw and trimmed data generated by *ngsShoRT* algorithms and other tools. **Table SA7-2.** Correctness of *Saccharomyces cerevisiae S288c de novo* genome assemblies of raw and trimmed data generated by *ngsShoRT* algorithms and other tools. **Table SA7-3.** Correctness of *Escherichia coli O157 H7 de novo* genome assemblies of raw and trimmed data generated by *ngsShoRT* algorithms and other tools. **Table SA8-1.** Summary of *Caenorhabditis elegans* reference-based assemblies of raw and trimmed data generated by *ngsShoRT* algorithms and other tools. **Table SA8-2.** Summary of *Saccharomyces cerevisiae S288c* reference-based assemblies of raw and trimmed data generated by *ngsShoRT* algorithms and other tools. **Table SA8-3.** Summary of *Escherichia coli O157 H7* reference-based assemblies of raw and trimmed data generated by *ngsShoRT* algorithms and other tools. Click here for file
