# Supplementary material for: Software for pre-processing Illumina next-generation sequencing short read sequences
Source: Source Code Biol Med. 2014 May 3;9:8. doi: 10.1186/1751-0473-9-8 (PMC4064128; doi:10.1186/1751-0473-9-8)
Supplement: Additional file 1: Figure S1 — Workflow and functional components of ngsShoRT software. Figure S2. Workflow of DBG-based de novo assembly and reference-based assembly evaluation experiments. Figure S3. Summary of trimmed data generated by ngsShoRT algorithms. Figure S4. Summary of trimmed data generated by ngsShoRT algorithms and other tools. Figure S5. Summary of de novo genome assemblies of raw and trimmed data generated by ngsShoRT algorithms. Figure S6. Correctness of de novo genome assemblies of raw and trimmed data generated by ngsShoRT algorithms. Figure S7. Summary of de novo genome assemblies of raw and trimmed data generated by ngsShoRT algorithms and other tools. Figure S8. Correctness of de novo assemblies of raw and trimmed data generated by ngsShoRT algorithms and other tools. Figure S9. Summary of reference-based assemblies of raw and trimmed data generated by ngsShoRT algorithms. Figure S10. Summary of reference-based assemblies of raw and trimmed data generated by ngsShoRT algorithms and other tools. [file 1751-0473-9-8-S1.pdf]

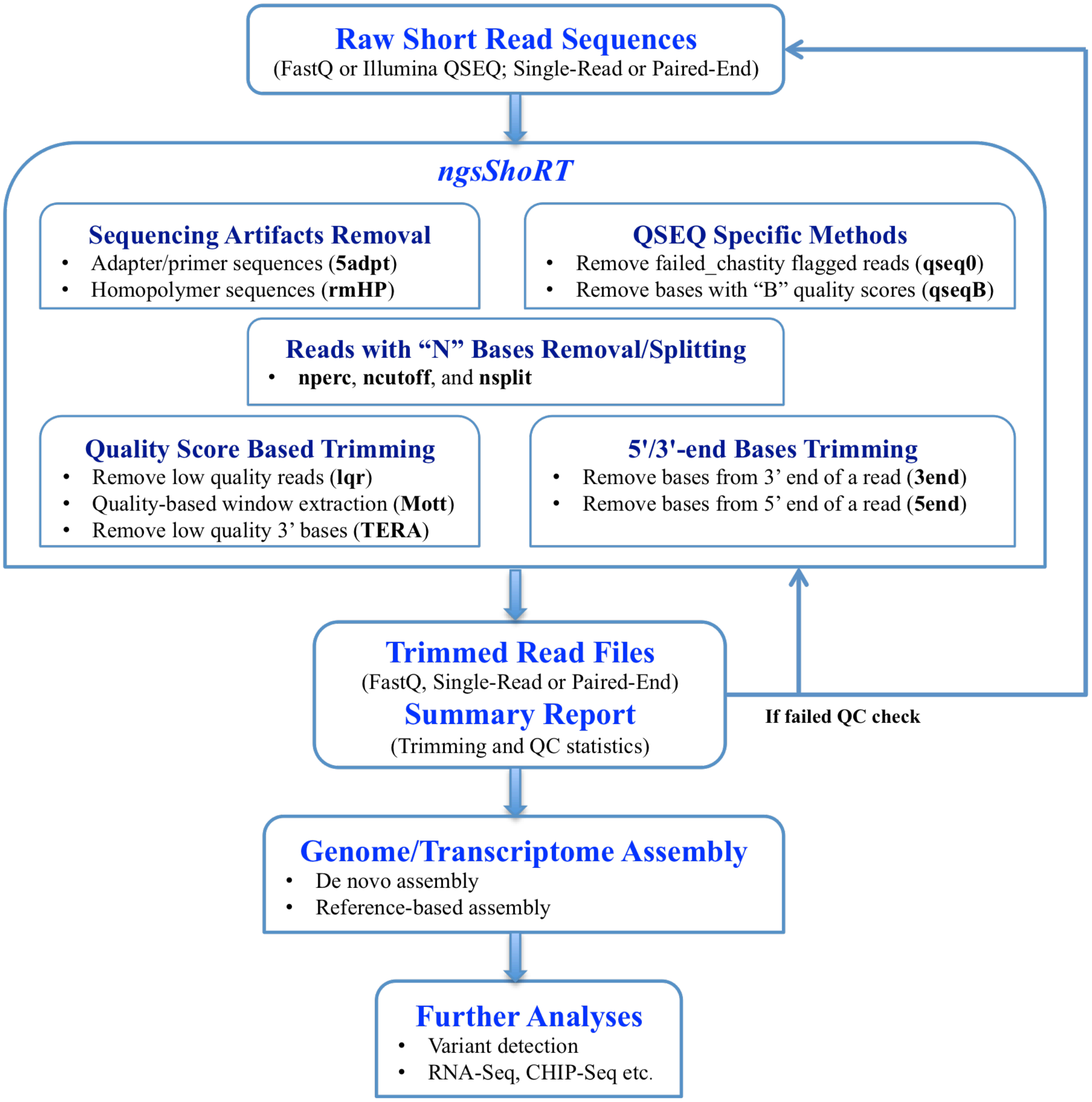

Figure S1. Workflow and functional components of *ngsShoRT* software

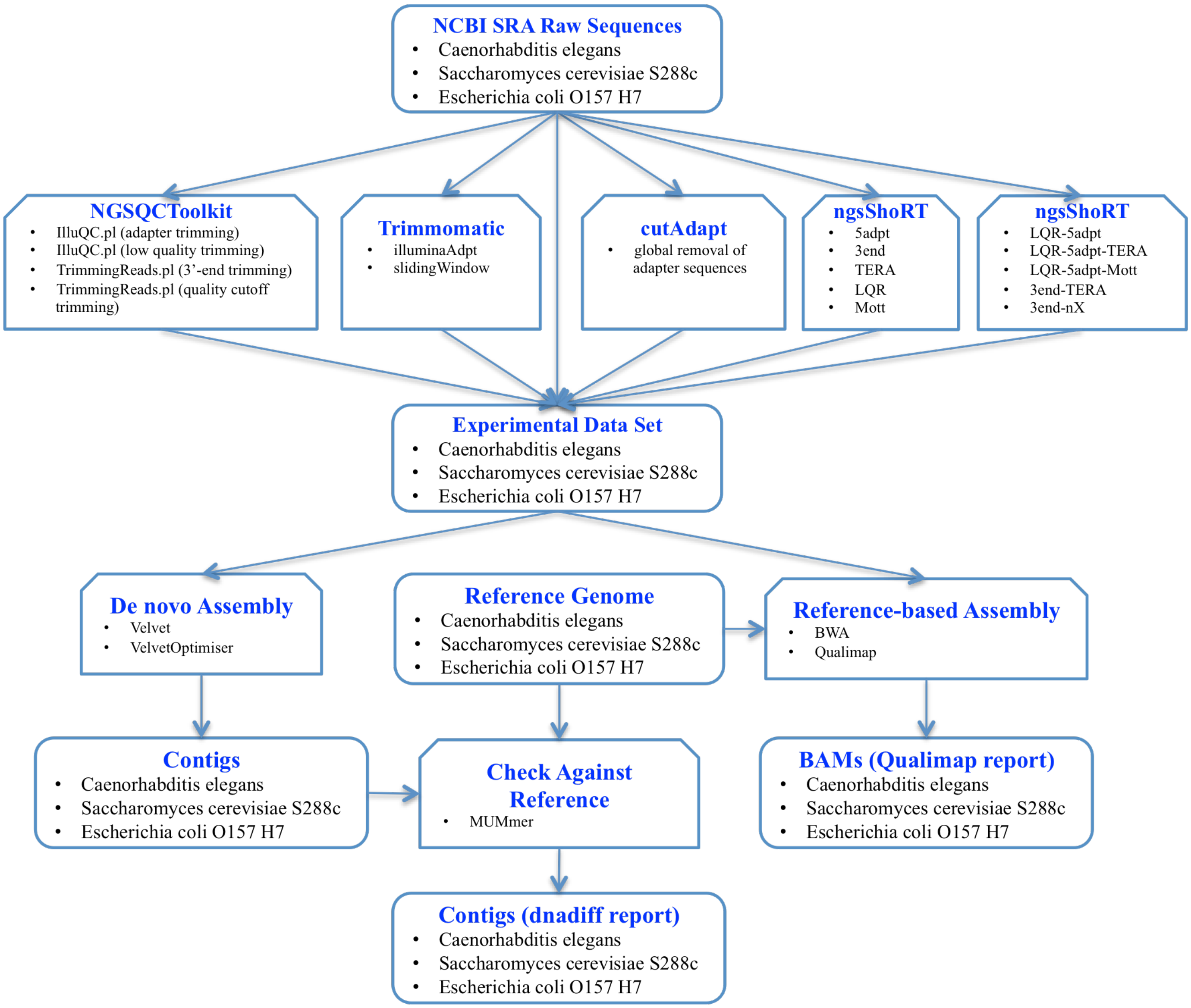

Figure S2. Workflow of DBG-based *de novo* assembly and reference-based assembly evaluation experiments

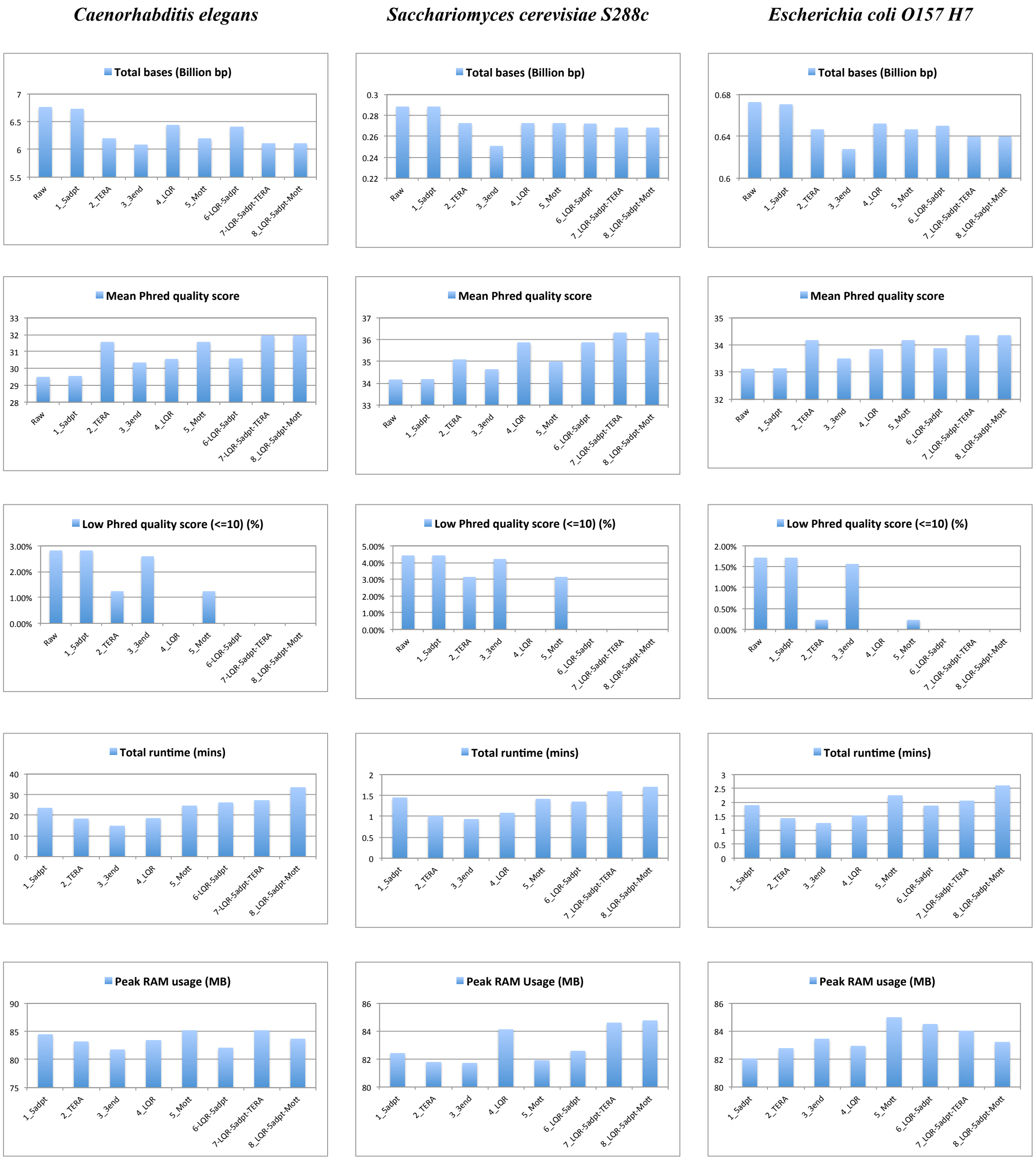

Figure S3. Summary of trimmed data generated by *ngsShoRT* algorithms

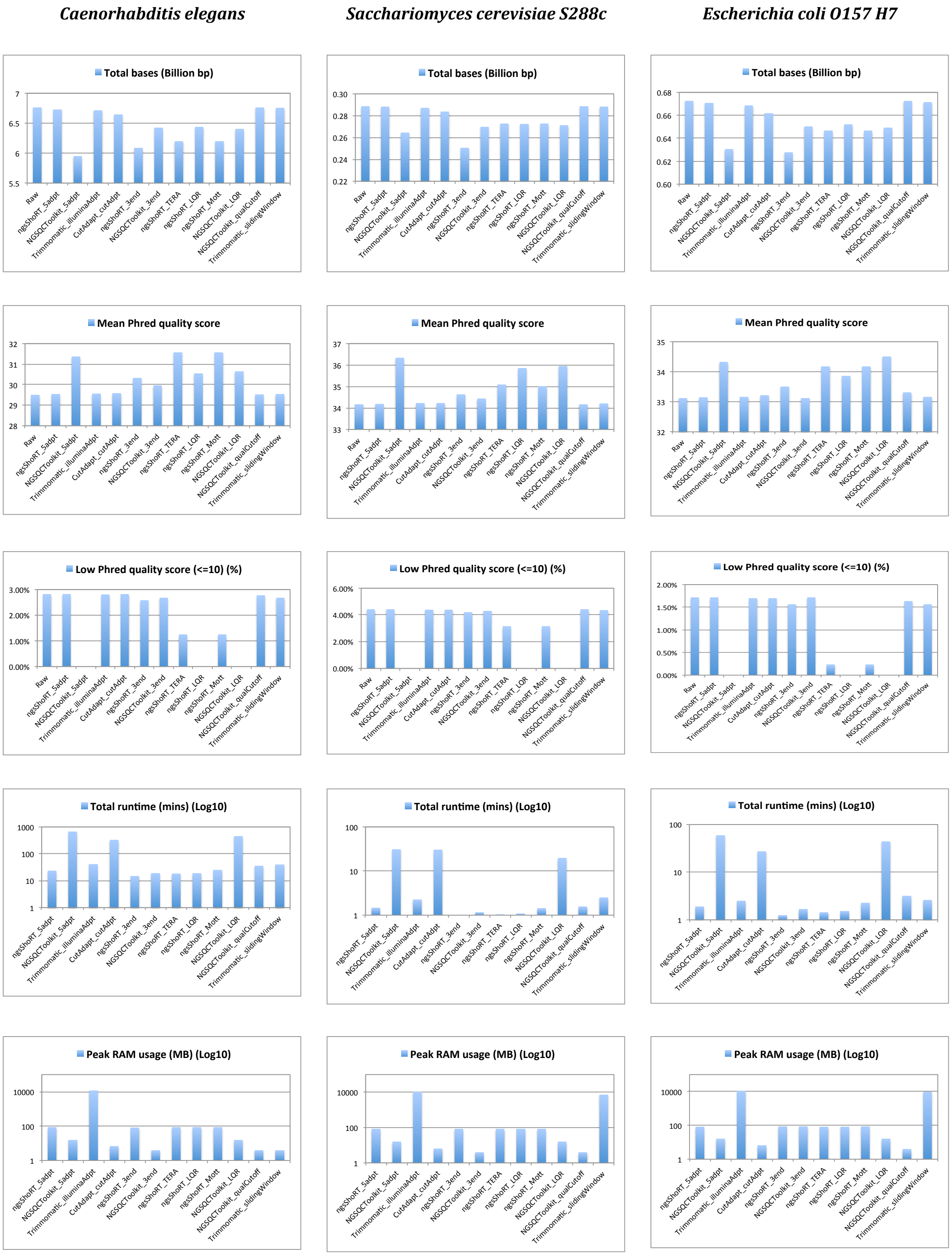

Figure S4. Summary of trimmed data generated by *ngsShoRT* algorithms and other tools

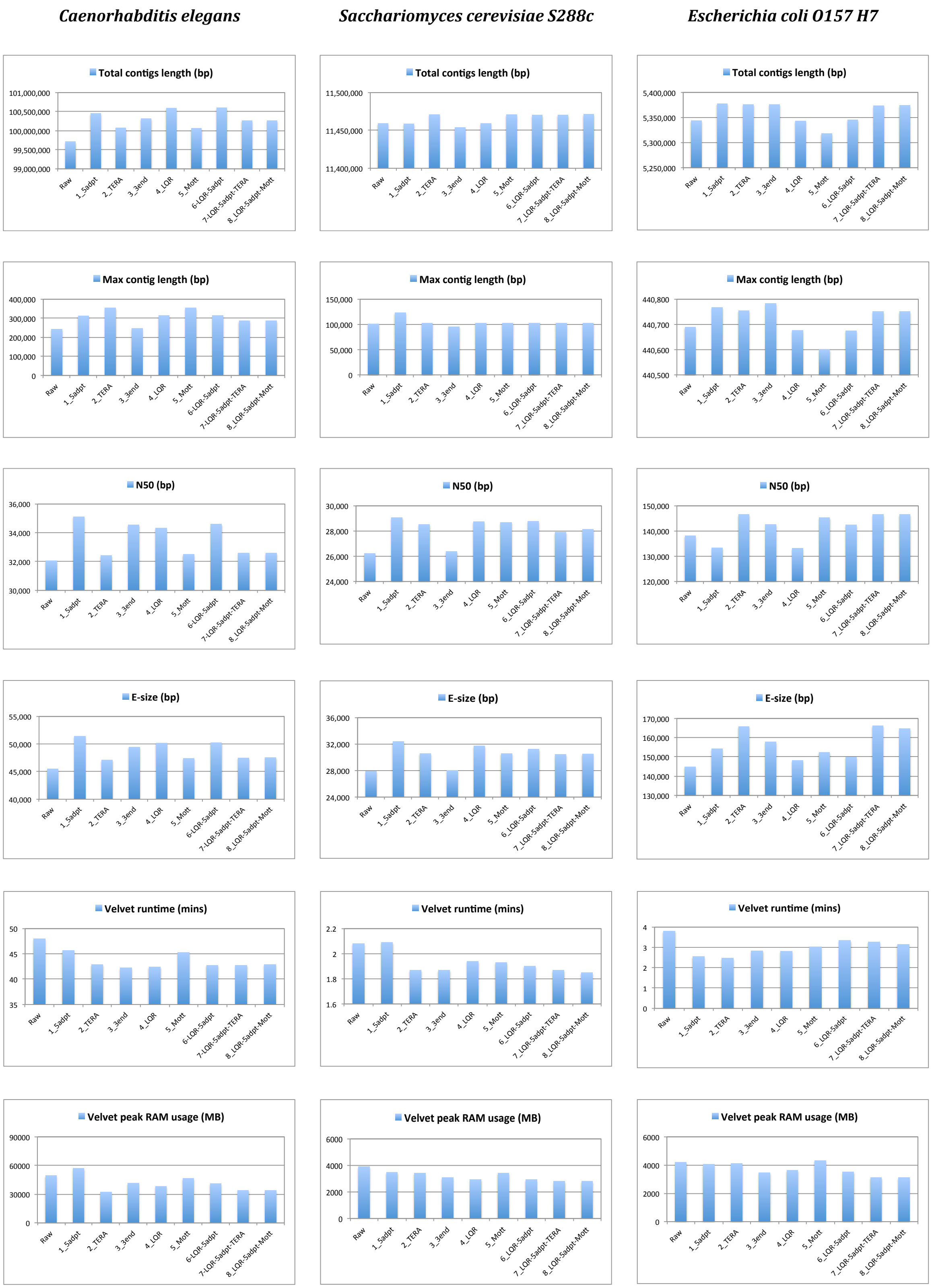

Figure S5. Summary of *de novo* genome assemblies of raw and trimmed data generated by *ngsShoRT* algorithms

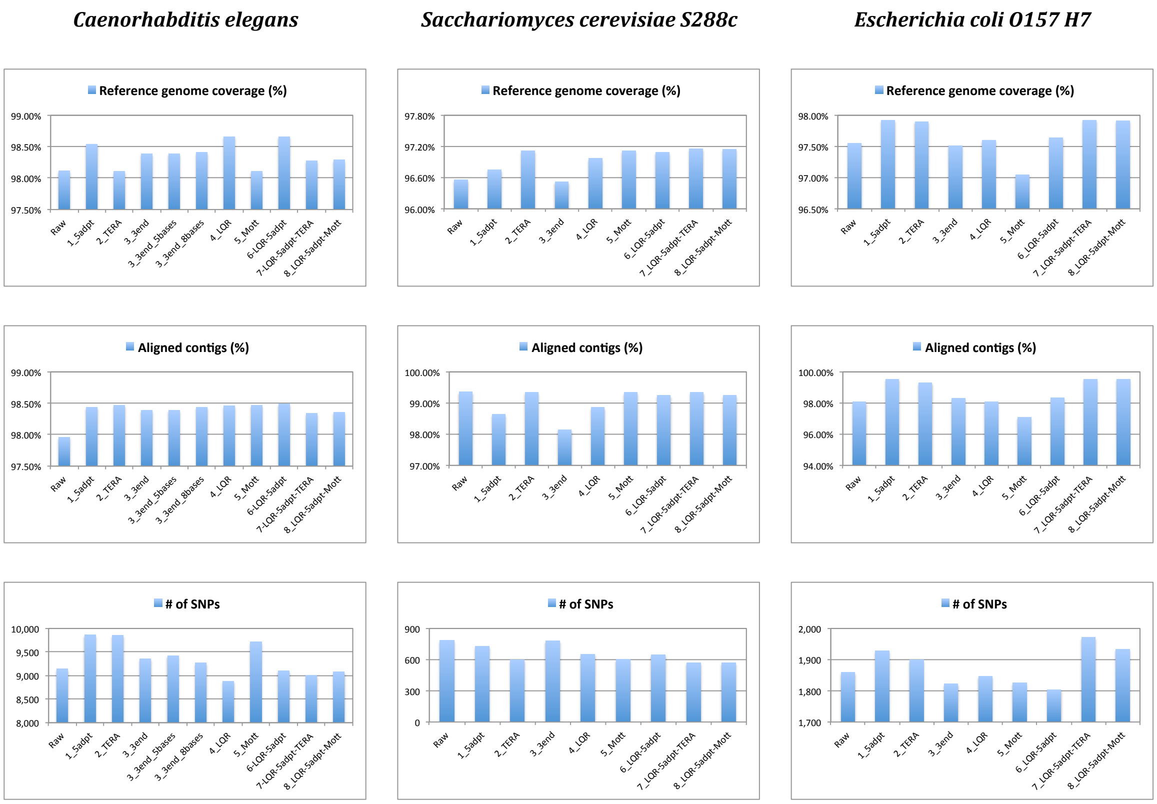

Figure S6. Correctness of *de novo* genome assemblies of raw and trimmed data generated by *ngsShoRT* algorithms

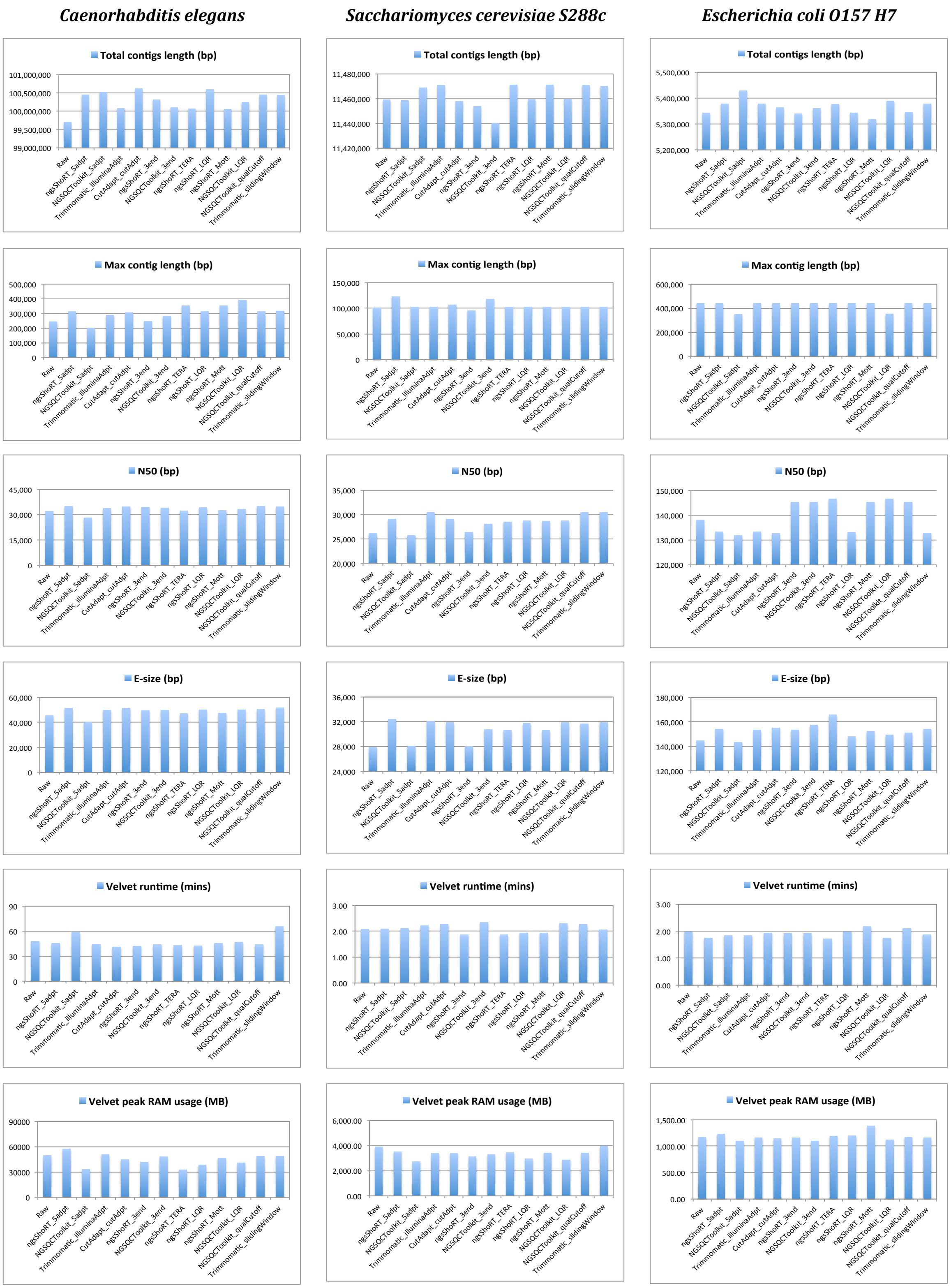

Figure S7. Summary of *de novo* genome assemblies of raw and trimmed data generated by *ngsShoRT* algorithms and other tools

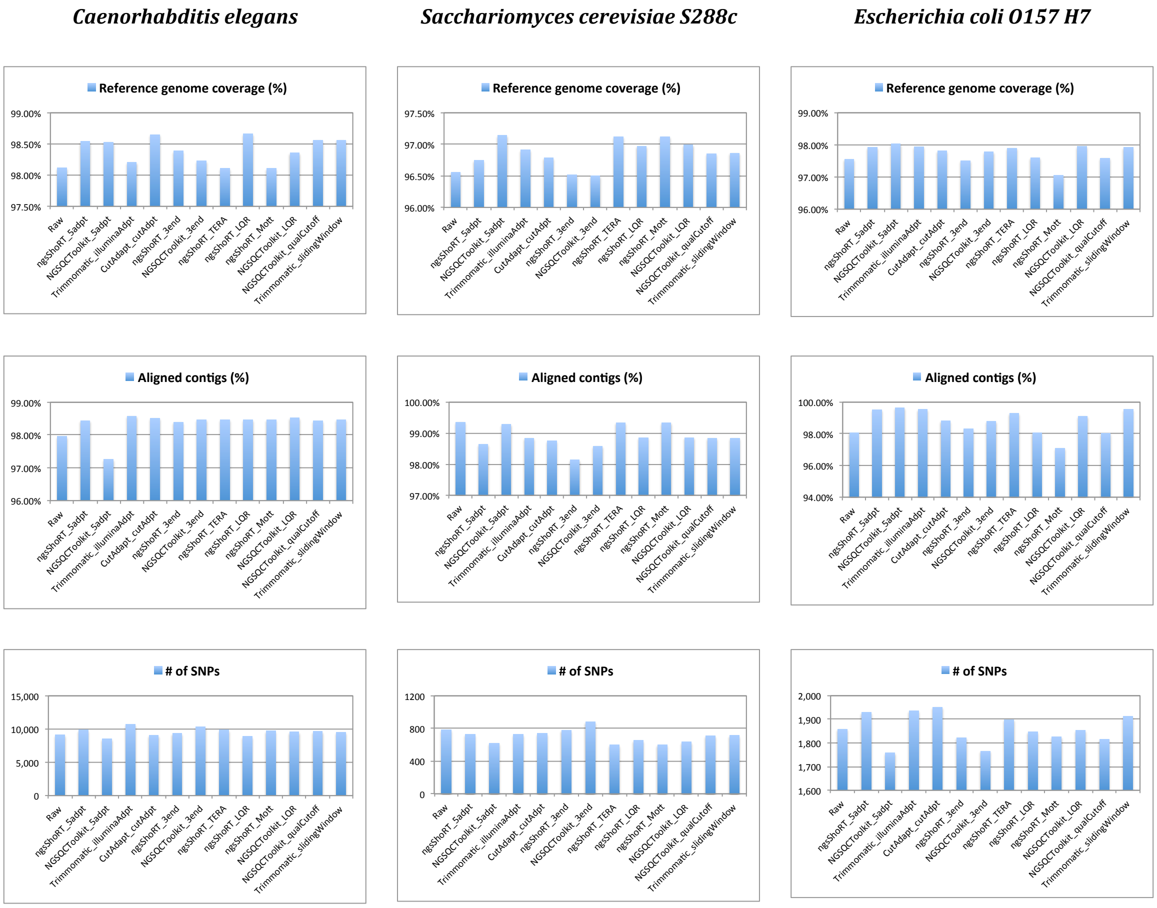

Figure S8. Correctness of *de novo* assemblies of raw and trimmed data generated by *ngsShoRT* algorithms and other tools

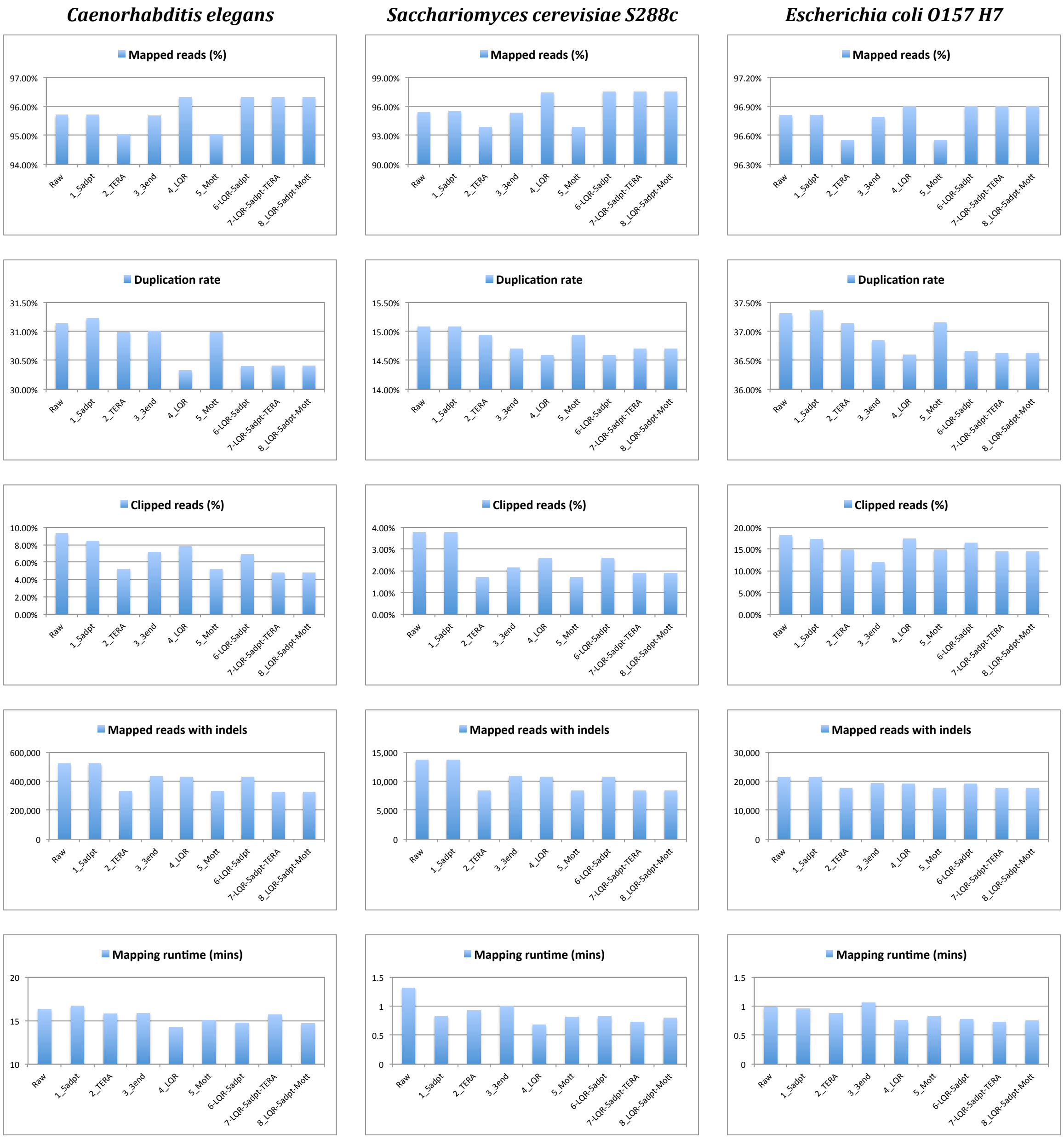

Figure S9. Summary of reference-based assemblies of raw and trimmed data generated by *ngsShoRT* algorithms

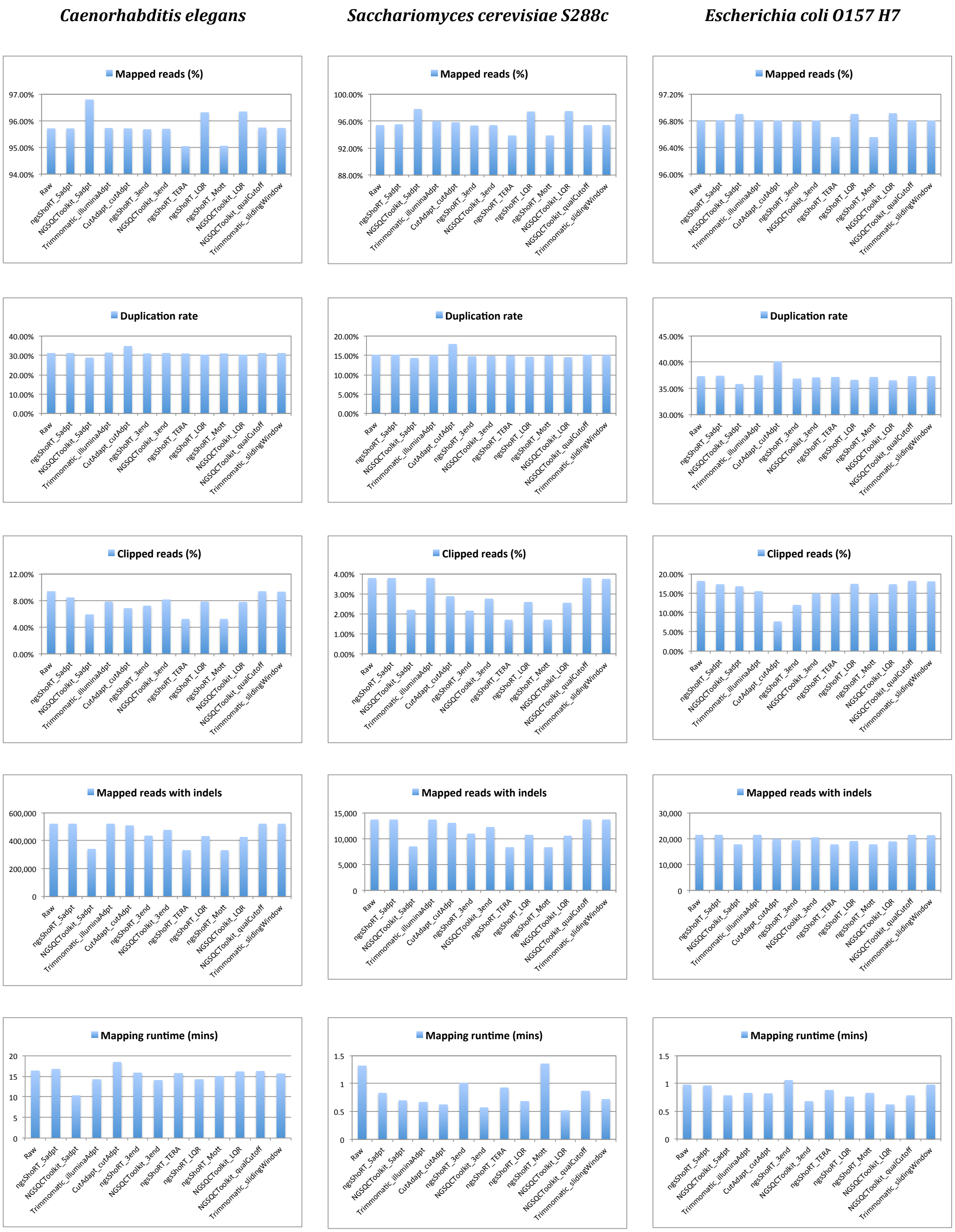

Figure S10. Summary of reference-based assemblies of raw and trimmed data generated by *ngsShoRT* algorithms and other tools
