# Supplementary material for: Software for pre-processing Illumina next-generation sequencing short read sequences
Source: Source Code Biol Med. 2014 May 3;9:8. doi: 10.1186/1751-0473-9-8 (PMC4064128; doi:10.1186/1751-0473-9-8)
Supplement: Additional file 2: Table SA1-1 — Summary of Caenorhabditis elegans trimmed data generated by ngsShoRT algorithms. Table SA1-2. Summary of Saccharomyces cerevisiae S288c trimmed data generated by ngsShoRT algorithms. Table SA1-3. Summary of Escherichia coli O157 H7 trimmed data generated by ngsShoRT algorithms. Table SA2-1. Summary of Caenorhabditis elegans de novo genome assemblies of raw and trimmed data generated by ngsShoRT algorithms. Table SA2-2. Summary of Saccharomyces cerevisiae S288c de novo genome assemblies of raw and trimmed data generated by ngsShoRT algorithms. Table SA2-3. Summary of Escherichia coli O157 H7 de novo genome assemblies of raw and trimmed data generated by ngsShoRT algorithms. Table SA3-1. Correctness of Caenorhabditis elegans de novo genome assemblies of raw and trimmed data generated by ngsShoRT algorithms. Table SA3-2. Correctness of Saccharomyces cerevisiae S288c de novo genome assemblies of raw and trimmed data generated by ngsShoRT algorithms. Table SA3-3. Correctness of Escherichia coli O157 H7 de novo genome assemblies of raw and trimmed data generated by ngsShoRT algorithms. Table SA4-1. Summary of Caenorhabditis elegans reference-based assemblies of raw and trimmed data generated by ngsShoRT algorithms. Table SA4-2. Summary of Saccharomyces cerevisiae S288c reference-based assemblies of raw and trimmed data generated by ngsShoRT algorithms. Table SA4-3. Summary of Escherichia coli O157 H7 reference-based assemblies of raw and trimmed data generated by ngsShoRT algorithms. [file 1751-0473-9-8-S2.pdf]

Table SA1-1 Summary of *Caenorhabditis elegans* trimmed data generated by *ngsShoRT* algorithms

**Times**	**1_5adpt**	**2_TERA**	**3_3end**	**4_LQR**	**5_Mott**	**6-LQR-5adpt**	**7-LQR-5adpt-TERA**	**8_LQR-5adpt-Mott**
**# threads**	32	32	32	32	32	32	32	32
**Trimming algorithm**	5adpt	tera	3end	lqr	mott	lqr, 5adpt	lqr, 5adpt, tera	lqr, 5adpt, mott
**Trimming algorithm parameters**	mrl: 21, list=Illumina adapter, action = ka, mp = 100	mrl: 21, avg = 2	mrl: 21, x=10	mrl: 21, lqs = 4, p = 50	mrl: 21, ml=0.6	mrl: 21, lqs = 4, p = 50, list=Illumina adapter, action = ka, mp = 100	mrl: 21, lqs = 4, p = 50, list=Illumina adapter, action = ka, mp = 100, avg=2	mrl: 21, lqs = 4, p = 50, list=Illumina adapter, action = ka, mp = 100, ml=0.6
**Mean Phred quality score**	29.53	31.57	30.33	30.55	31.57	30.58	31.96	31.96
**Low Phred quality score (<=10)**	1,899,444 (2.81%)	835,813 (1.24%)	1,741,324 (2.58%)	256 (0.00%)	835,825 (1.24%)	252 (0.00%)	105 (0.00%)	105 (0.00%)
**Total runtime (mins)**	23.51	18.31	14.76	18.58	24.65	26.15	27.3	33.56
**Peak thread runtime (mins)**	9.35	8.05	6.05	8.58	15.31	12.4	13.6	20.25
**Merging runtime (mins)**	14.03	10.18	8.6	10.18	9.21	13.61	13.56	13.18
**Peak RAM usage (MB)**	84.5	83.17	81.73	83.42	85.21	82.08	85.2	83.7
**Total sequences (paired)**	33,804,923	33,808,546	33,808,546	31,136,730	33,808,546	31,135,867	31,135,867	31,135,867
**Total bases (paired)**	6,727,619,415	6,198,092,855	6,085,538,280	6,227,346,000	6,198,004,714	6,195,604,058	5,894,323,879	5,894,186,634
**Total sequences (single)**	2,748	0	0	2,103,478	0	2,103,637	2,103,637	2,103,637
**Total bases (single)**	274,511	0	0	210,347,800	0	210,363,569	210,363,569	210,363,569
**Total bases**	6,727,893,926	6,198,092,855	6,085,538,280	6,437,693,800	6,198,004,714	6,405,967,627	6,104,687,448	6,104,550,203

Table SA1-2 Summary of *Saccharomyces cerevisiae S288c* trimmed data generated by *ngsShoRT* algorithms

**Times**	**1_5adpt**	**2_TERA**	**3_3end**	**4_LQR**	**5_Mott**	**6-LQR-5adpt**	**7-LQR-5adpt-TERA**	**8_LQR-5adpt-Mott**
**# threads**	32	32	32	32	32	32	32	32
**Trimming algorithm**	5adpt	tera	3end	lqr	mott	lqr, 5adpt	lqr, 5adpt, tera	lqr, 5adpt, mott
**Trimming algorithm parameters**	mrl: 21, list=Illumina adapter, action = ka, mp = 100	mrl: 21, avg = 2	mrl: 21, x=10	mrl: 21, lqs = 4, p = 50	mrl: 21, ml=0.6	mrl: 21, lqs = 4, p = 50, list=Illumina adapter, action = ka, mp = 100	mrl: 21, lqs = 4, p = 50, list=Illumina adapter, action = ka, mp = 100, avg=2	mrl: 21, lqs = 4, p = 50, list=Illumina adapter, action = ka, mp = 100, ml=0.6
**Mean Phred quality score**	34.18	35.08	34.63	35.86	35	35.87	36.32	36.32
**Low Phred quality score (<=10)**	167,587 (4.42%)	119,114 (3.14%)	159,108 (4.19%)	4 (0.00%)	119,114 (3.14%)	4 (0.00%)	3 (0.00%)	3 (0.00%)
**Total runtime (mins)**	1.45	1.01	0.93	1.08	1.42	1.35	1.6	1.7
**Peak hread runtime (mins)**	0.7	0.45	0.4	0.53	0.88	0.61	0.83	1.01
**Merging runtime (mins)**	0.7	0.56	0.53	0.55	0.53	0.73	0.76	0.68
**Peak RAM usage (MB)**	82.43	81.78	81.7	84.12	81.91	82.59	84.61	84.76
**Total sequences (paired)**	1,894,251	1,898,259	1,898,259	1,740,787	1,898,259	1,737,001	1,737,001	1,737,001
**Total bases (paired)**	287,920,526	272,556,467	250,570,188	264,599,624	272,556,067	264,020,941	260,037,396	260,037,396
**Total sequences (single)**	4,008	0	0	102,619	0	106,405	106,405	106,405
**Total bases (single)**	304,608	0	0	7,799,044	0	8,086,780	8,086,780	8,086,780
**Total bases**	288,225,134	272,556,467	250,570,188	272,398,668	272,556,067	272,107,721	268,124,176	268,124,176

Table SA1-3 Summary of *Escherichia coli O157 H7* trimmed data generated by *ngsShoRT* algorithms

**Times**	**1_5adpt**	**2_TERA**	**3_3end**	**4_LQR**	**5_Mott**	**6-LQR-5adpt**	**7-LQR-5adpt-TERA**	**8_LQR-5adpt-Mott**
**# threads**	32	32	32	32	32	32	32	32
**Trimming algorithm**	5adpt	tera	3end	lqr	mott	lqr, 5adpt	lqr, 5adpt, tera	lqr, 5adpt, mott
**Trimming algorithm parameters**	mrl: 21, list=Illumina adapter, action = ka, mp = 100	mrl: 21, avg = 2	mrl: 21, x=10	mrl: 21, lqs = 4, p = 50	mrl: 21, ml=0.6	mrl: 21, lqs = 4, p = 50, list=Illumina adapter, action = ka, mp = 100	mrl: 21, lqs = 4, p = 50, list=Illumina adapter, action = ka, mp = 100, avg=2	mrl: 21, lqs = 4, p = 50, list=Illumina adapter, action = ka, mp = 100, ml=0.6
**Mean Phred quality score**	33.14	34.17	33.5	33.85	34.17	33.87	34.35	34.35
**Low Phred quality score (<=10)**	76,569 (1.71%)	10,492 (0.23%)	69,820 (1.56%)	0 (0.00%)	10492 (0.23%)	0 (0.00%)	0 (0.00%)	0 (0.00%)
**Total runtime (mins)**	1.9	1.43	1.25	1.53	2.25	1.88	2.06	2.6
**Peak thread runtime (mins)**	0.9	0.67	0.52	0.78	1.51	1.03	1.18	1.73
**Merging runtime (mins)**	0.98	0.76	0.73	0.73	0.71	0.83	0.86	0.86
**Peak RAM usage (MB)**	82.02	82.76	83.44	82.95	84.98	84.5	84.01	83.21
**Total sequences (paired)**	2,241,777	2,241,778	2,241,778	2,113,808	2,241,778	2,113,807	2,113,807	2,113,807
**Total bases (paired)**	670,699,844	646,423,163	627,697,840	634,142,400	646,418,288	632,339,583	621,772,745	621,769,058
**Total sequences (single)**	1	0	0	118,827	0	118,828	118,828	118,828
**Total bases (single)**	150	0	0	17,824,050	0	17,824,200	17,824,200	17,824,200
**Total bases (Million)**	670,699,994	646,423,163	627,697,840	651,966,450	646,418,288	650,163,783	639,596,945	639,593,258

Table SA2-1 Summary of *Caenorhabditis elegans de novo* genome assemblies of raw and trimmed data generated by *ngsShoRT* algorithms

DataSet	Raw	1_5adpt	2_TERA	3_3end	4_LQR	5_Mott	6-LQR-5adpt	7-LQR-5adpt-TERA	8_LQR-5adpt-Mott
**VelvetOptimiser K-mer range**	21 to 71, step 2	21 to 71, step 2	21 to 71, step 2	21 to 71, step 2	21 to 71, step 2	21 to 71, step 2	21 to 71, step 2	21 to 71, step 2	21 to 71, step 2
**Optimal K-mer**	59	57	53	55	59	53	59	55	55
**Total contigs length (bp)**	99,712,970	100,450,601	100,071,634	100,319,545	100,597,965	100,060,740	100,608,320	100,270,458	100,267,229
**Total # contigs**	10,789	10,404	10,776	10,383	10,549	10,755	10,573	10,792	10,781
**Max contig length (bp)**	242,599	313,669	354,328	246,594	313,984	354,102	313,982	286,554	286,554
**E-size (bp)**	45,484	51,429	47,096	49,483	50,183	47,424	50,225	47,516	47,577
**N25 (bp)**	62,833	67,636	62,934	66,635	67,555	63,244	67,555	64,578	64,665
**N50 (bp)**	32,064	35,124	32,417	34,566	34,320	32,509	34,603	32,606	32,606
**N75 (bp)**	14,163	15,640	14,589	15,704	15,429	14,684	15,391	14,440	14,444
**N90 (bp)**	5,637	6,187	5,709	6,093	6,059	5,713	6,039	5,719	5,713
**GC content (%)**	35.78	35.88	35.8	35.86	35.86	35.8	35.86	35.79	35.79
**Velveth runtime (mins)**	13.85	13.76	11.23	10.76	10.35	11.5	11	10.7	10.48
**Velveth peak RAM usage (MB)**	33292.17	41054.26	16867.36	26148.76	23361.7	31367.98	25280.1	18661.54	18657.1
**Velvetg runtime (mins)**	34.18	31.91	31.67	31.52	32.06	33.8	31.76	32.08	32.46
**Velvetg peak RAM usage (MB)**	16227.09	16250.76	15450.44	15487.39	15064.87	15305.32	15900.02	15584.98	15586.46
**Velvet runtime (mins)**	48.03	45.67	42.9	42.28	42.41	45.3	42.76	42.78	42.94
**Velvet peak RAM usage (MB)**	49519.26	57305.02	32317.8	41636.15	38426.57	46673.3	41180.12	34246.52	34243.56

Table SA2-2 Summary of *Saccharomyces cerevisiae S288c de novo* genome assemblies of raw and trimmed data generated by *ngsShoRT* algorithms

DataSet	Raw	1_5adpt	2_TERA	3_3end	4_LQR	5_Mott	6-LQR-5adpt	7-LQR-5adpt-TERA	8_LQR-5adpt-Mott
**VelvetOptimiser K-mer range**	21 to 71, step 2	21 to 71, step 2	21 to 71, step 2	21 to 71, step 2	21 to 71, step 2	21 to 71, step 2	21 to 71, step 2	21 to 71, step 2	21 to 71, step 2
**Optimal K-mer**	31	31	33	31	31	33	33	33	33
**Total contigs length (bp)**	11,459,063	11,458,557	11,471,167	11,453,735	11,459,492	11,471,030	11,470,227	11,470,472	11,471,278
**Total # contigs**	1097	1,032	1,057	1,135	1,053	1,057	1,062	1,068	1,064
**Max contig length (bp)**	101,217	123,093	102,654	95,726	102,663	102,654	102,651	102,652	102,652
**E-size (bp)**	27,917	32,412	30,607	28,027	31,744	30,610	31,238	30,460	30,543
**N25 (bp)**	39,313	46,832	45,306	39,910	46,227	45,306	47,090	45,309	45,309
**N50 (bp)**	26,221	29,080	28,539	26,386	28,753	28,692	28,769	27,918	28,152
**N75 (bp)**	14,483	16,450	15,439	13,864	15,642	15,439	15,515	14,903	14,903
**N90 (bp)**	7,100	8,320	7,384	6,866	7,828	7,708	7,285	7,180	7,288
**GC content (%)**	38.1	38.07	38.06	38.06	38.08	38.06	38.07	38.06	38.06
**Velveth runtime (mins)**	0.5	0.47	0.42	0.42	0.42	0.43	0.42	0.42	0.42
**Velveth peak RAM usage (MB)**	2386.38	1953.91	1972.8	1701.79	1475.32	1972.27	1475.77	1370.6	1372.48
**Velvetg runtime (mins)**	1.58	1.62	1.45	1.45	1.52	1.5	1.48	1.45	1.43
**Velvetg peak RAM usage (MB)**	1528.89	1541.94	1457.18	1401.07	1474.82	1457.29	1454.1	1440.28	1439.76
**Velvet runtime (mins)**	2.08	2.09	1.87	1.87	1.94	1.93	1.9	1.87	1.85
**Velvet peak RAM usage (MB)**	3915.27	3495.85	3429.98	3102.86	2950.14	3429.56	2929.87	2810.88	2812.24

Table SA2-3 Summary of *Escherichia coli O157 H7 de novo* genome assemblies of raw and trimmed data generated by *ngsShoRT* algorithms

DataSet	Raw	1_5adpt	2_TERA	3_3end	4_LQR	5_Mott	6-LQR-5adpt	7-LQR-5adpt-TERA	8_LQR-5adpt-Mott
**VelvetOptimiser K-mer range**	21 to 101, step 2	21 to 101, step 2	21 to 101, step 2	21 to 101, step 2	21 to 101, step 2	21 to 101, step 2	21 to 101, step 2	21 to 101, step 2	21 to 101, step 2
**Optimal K-mer**	65	83	81	81	63	53	63	79	79
**Total contigs length (bp)**	5,343,509	5,377,817	5,376,030	5,376,062	5,343,371	5,318,483	5,345,366	5,373,725	5,374,022
**Total # contigs**	417	429	426	433	415	413	419	428	429
**Max contig length (bp)**	440,689	440,768	440,755	440,783	440,676	440,600	440,675	440,751	440,751
**E-size (bp)**	144,862	154,338	165,801	157,755	148206	152,471	149,904	166,198	164,754
**N25 (bp)**	206,077	201,538	269,539	217,745	217,634	219,582	217,634	269,465	238,346
**N50 (bp)**	138,164	133,303	146,563	142,592	133,173	145,302	142,431	146,559	146,559
**N75 (bp)**	55,036	58,448	65,903	61,027	55,026	49,914	55,026	65,899	63,153
**N90 (bp)**	19,524	19,668	25,042	21,821	21,336	20,376	21,336	26,226	26,226
**GC content (%)**	50.24	50.24	50.25	50.25	50.24	50.23	50.24	50.25	50.25
**Velveth runtime (mins)**	1.82	0.8	0.75	1.15	0.83	0.86	1.32	1.55	1.46
**Velveth peak RAM usage (MB)**	3039.85	2823.38	2921.06	2318.01	2434.88	2932.83	2327.39	1935.74	1935.88
**Velvetg runtime (mins)**	1.98	1.75	1.72	1.68	1.98	2.18	2.02	1.72	1.7
**Velvetg peak RAM usage (MB)**	1171.93	1228.18	1192.27	1149.82	1203.38	1383.91	1201.78	1181.9	118.15
**Velvet runtime (mins)**	3.8	2.55	2.47	2.83	2.81	3.04	3.34	3.27	3.16
**Velvet peak RAM usage (MB)**	4211.78	4051.56	4113.33	3467.83	3638.26	4316.74	3529.17	3117.64	3117.03

Table SA3-1 Correctness of *Caenorhabditis elegans de novo* genome assemblies of raw and trimmed data generated by *ngsShoRT* algorithms

DataSet	Raw	1_5adpt	2_TERA	3_3end	4_LQR	5_Mott	6-LQR-5adpt	7-LQR-5adpt-TERA	8_LQR-5adpt-Mott
**Reference genome size**	100,286,401	100,286,401	100,286,401	100,286,401	100,286,401	100,286,401	100,286,401	100,286,401	100,286,401
**Reference genome coverage**	98,403,841 (98.12%)	98,822,655 (98.54%)	98,392,719 (98.11%)	98,669,067 (98.39%)	98,939,675 (98.66%)	98,393,760 (98.11%)	98,944,939 (98.66%)	98,563,710 (98.28%)	98,569,358 (98.29%)
**Total contigs**	10,789	10,404	10,776	10,352	10,549	10,755	10,573	10,792	10,781
**Aligned contigs**	10,569 (97.96%)	10,242 (98.44%)	10,611 (98.47%)	10,185 (98.39%)	10,387 (98.46%)	10,590 (98.47%)	10,413 (98.49%)	10,613 (98.34%)	10,604 (98.36%)
**Avg identity (%) (1-1 mapping)**	99.96	99.96	99.94	99.95	99.96	99.94	99.96	99.94	99.94
**Avg identity (%) (m-m mapping)**	99.85	99.85	99.84	99.85	99.85	99.84	99.85	99.84	99.84
**TotalSNPs**	9,140	9,868	9,860	9,358	8,882	9,717	9,105	9,008	9,076

Table SA3-2 Correctness of *Saccharomyces cerevisiae S288c de novo* genome assemblies of raw and trimmed data generated by *ngsShoRT* algorithms

**DataSet**	**Raw**	**1_5adpt**	**2_TERA**	**3_3end**	**4_LQR**	**5_Mott**	**6-LQR-5adpt**	**7-LQR-5adpt-TERA**	**8_LQR-5adpt-Mott**
**Reference genome size**	12,157,105	12,157,105	12,157,105	12,157,105	12,157,105	12,157,105	12,157,105	12,157,105	12,157,105
**Reference genome coverage**	11,738,621 (96.56%)	11,761,633 (96.75%)	11,809,050 (97.14%)	11,781,549 (96.91%)	11766257 (96.79%)	11,734,644 (96.52%)	11,731,863 (96.50%)	11,806,630 (97.12%)	11,789,294 (96.97%)
**Total contigs**	1,097	1,032	1,124	1,035	1,040	1,135	1,054	1,057	1,053
**Aligned contigs**	1,090 (99.36%)	1,018 (98.64%)	1,116 (99.29%)	1.023 (98.84%)	1.027 (98.75%)	1,114 (98.15%)	1,039 (98.58%)	1,050 (99.34%)	1,041 (98.86%)
**Avg identity (%) (1-1 mapping)**	99.96	99.97	99.97	99.97	99.97	99.95	99.97	99.96	99.97
**Avg identity (%) (m-m mapping)**	99.95	99.95	99.95	99.95	99.95	99.94	99.95	99.94	99.96
**TotalSNPs**	786	730	621	729	739	781	882	598	654

Table SA3-3 Correctness of *Escherichia coli O157 H7 de novo* genome assemblies of raw and trimmed data generated by *ngsShoRT* algorithms

DataSet	Raw	1_5adpt	2_TERA	3_3end	4_LQR	5_Mott	6-LQR-5adpt	7-LQR-5adpt-TERA	8_LQR-5adpt-Mott
**Reference genome size**	5,498,450	5,498,450	5,498,450	5,498,450	5,498,450	5,498,450	5,498,450	5,498,450	5,498,450
**Reference genome coverage**	5363955 (97.55%)	5384193 (97.92%)	5,389,948 (98.03%)	5,384,971 (97.94%)	5,378,185 (97.81%)	5361630 (97.51%)	5,376,324 (97.78%)	5383225 (97.90%)	5366345 (97.60%)
**Total contigs**	417	429	565	432	425	413	416	426	415
**Aligned contigs**	409 (98.08%)	427 (99.53%)	563 (99.65%)	430 (99.54%)	420 (98.82%)	406 (98.31%)	411 (98.80%)	423 (99.30%)	407 (98.07%)
**Avg identity (%) (1-1 mapping)**	99.95	99.95	99.96	99.95	99.95	99.95	99.96	99.95	99.96
**Avg identity (%) (m-m mapping)**	99.9	99.89	99.9	99.89	99.89	99.9	99.9	99.9	99.9
**TotalSNPs**	1,859	1,929	1,758	1,936	1,952	1,823	1,766	1,899	1,847

Table SA4-1 Summary of *Caenorhabditis elegans* reference-based assemblies of raw and trimmed data generated by *ngsShoRT* algorithms

DataSet	Raw	1_5adpt	2_TERA	3_3end	4_LQR	5_Mott	6-LQR-5adpt	7-LQR-5adpt-TERA	8_LQR-5adpt-Mott
**Reference genome size**	100,286,401	100,286,401	100,286,401	100,286,401	100,286,401	100,286,401	100,286,401	100,286,401	100,286,401
**Number of reads**	68,029,090	68,024,577	67,948,294	67,901,888	64,751,598	67,948,294	64,750,021	64,712,569	64,712,569
**Mapped reads**	65,108,539 / 95.71%	65,107,835 / 95.71%	64,580,965 / 95.04%	64,966,762 / 95.68%	62,368,674 / 96.32%	64,582,097 / 95.05%	62,368,664 / 96.32%	62,326,365 / 96.31%	62,326,364 / 96.31%
**Unmapped reads**	2,920,551 / 4.29%	2,916,742 / 4.29%	3,367,329 / 4.96%	2,935,126 / 4.32%	2,382,924 / 3.68%	3,366,197 / 4.95%	2,381,357 / 3.68%	2,386,204 / 3.69%	2,386,205 / 3.69%
**Paired reads**	65,108,539 / 95.71%	65,107,653 / 95.71%	64,580,965 / 95.04%	64,966,762 / 95.68%	60,429,886 / 93.33%	64,582,097 / 95.05%	60,429,873 / 93.33%	60,387,574 / 93.32%	60,387,573 / 93.32%
**Mapped reads, only first in pair**	32,603,951 / 47.93%	32,603,560 / 47.93%	32,412,953 / 47.7%	32,535,473 / 47.92%	30,206,558 / 46.65%	32,414,026 / 47.7%	30,206,568 / 46.65%	30,186,321 / 46.65%	30,186,320 / 46.65%
**Mapped reads, only second in pair**	32,504,588 / 47.78%	32,504,093 / 47.78%	32,168,012 / 47.34%	32,431,289 / 47.76%	30,223,328 / 46.68%	32,168,071 / 47.34%	30,223,305 / 46.68%	30,201,253 / 46.67%	30,201,253 / 46.67%
**Mapped reads, both in pair**	64,782,848 / 95.23%	64,782,175 / 95.23%	64,044,875 / 94.26%	64,631,660 / 95.18%	60,307,426 / 93.14%	64,045,411 / 94.26%	60,307,390 / 93.14%	60,260,972 / 93.12%	60,260,972 / 93.12%
**Mapped reads, singletons**	325,691 / 0.48%	325,478 / 0.48%	536,090 / 0.79%	335,102 / 0.49%	122,460 / 0.19%	536,686 / 0.79%	122,483 / 0.19%	126,602 / 0.2%	126,601 / 0.2%
**Read min/max/mean length**	30 / 100 / 99.64	21 / 100 / 99.15	21 / 100 / 91.41	30 / 90 / 89.78	30 / 100 / 99.65	21 / 100 / 91.41	21 / 100 / 99.17	21 / 100 / 94.54	21 / 100 / 94.54
**Clipped reads**	6,368,951 / 9.36%	5,746,286 / 8.45%	3,555,657 / 5.23%	4,881,239 / 7.19%	5,057,053 / 7.81%	3,552,831 / 5.23%	4,462,053 / 6.89%	3,093,114 / 4.78%	3,092,255 / 4.78%
**Duplication rate**	31.14%	31.22%	30.99%	31.01%	30.33%	30.99%	30.40%	30.41%	30.41%
**GC (%)**	34.15%	34.15%	33.74%	34.09%	33.98%	33.74%	33.98%	33.70%	33.70%
**Total reads with indels**	521,946	522,212	330,934	433,970	431,268	330,864	431,385	327,210	327,313
**Insertions**	211,101	211,200	129,093	175,048	171,387	128,999	171,450	128,402	128,471
**Deletions**	310,845	311,012	201,841	258,922	259,881	201,865	259,935	198,808	198,842
**Mean insert size**	244.42	244.27	243.48	244.14	243.82	243.47	243.82	243.26	243.26
**Median insert size**	252	252	252	252	251	252	251	251	251
**Mapping runtime (mins)**	16.35	16.71	15.8	15.88	14.28	15.05	14.75	15.7	14.7

Table SA4-2 Summary of *Saccharomyces cerevisiae S288c* reference-based assemblies of raw and trimmed data generated by *ngsShoRT* algorithms

DataSet	Raw	1_5adpt	2_TERA	3_3end	4_LQR	5_Mott	6-LQR-5adpt	7-LQR-5adpt-TERA	8_LQR-5adpt-Mott
**Reference genome size**	12,157,105	12,157,105	12,157,105	12,157,105	12,157,105	12,157,105	12,157,105	12,157,105	12,157,105
**Number of reads**	3,803,781	3,799,773	3,802,165	3,800,411	3,590,603	3,802,165	3,586,817	3,586,640	3,586,640
**Mapped reads**	3,628,753 / 95.4%	3,628,737 / 95.5%	3,568,113 / 93.84%	3,623,766 / 95.35%	3,498,316 / 97.43%	3,568,113 / 93.84%	3,498,300 / 97.53%	3,498,053 / 97.53%	3,498,053 / 97.53%
**Unmapped reads**	175,028 / 4.6%	171,036 / 4.5%	234,052 / 6.16%	176,645 / 4.65%	92,287 / 2.57%	234,052 / 6.16%	88,517 / 2.47%	88,587 / 2.47%	88,587 / 2.47%
**Paired reads**	3,628,753 / 95.4%	3,628,737 / 95.5%	3,568,113 / 93.84%	3,623,766 / 95.35%	3,407,454 / 94.9%	3,568,113 / 93.84%	3,407,438 / 95%	3,407,191 / 95%	3,407,191 / 95%
**Mapped reads, only first in pair**	1,805,405 / 47.46%	1,805,397 / 47.51%	1,778,833 / 46.78%	1,804,231 / 47.47%	1,702,837 / 47.42%	1,778,833 / 46.78%	1,702,829 / 47.47%	1,702,734 / 47.47%	1,702,734 / 47.47%
**Mapped reads, only second in pair**	1,823,348 / 47.94%	1,823,340 / 47.99%	1,789,280 / 47.06%	1,819,535 / 47.88%	1,704,617 / 47.47%	1,789,280 / 47.06%	1,704,609 / 47.52%	1,704,457 / 47.52%	1,704,457 / 47.52%
**Mapped reads, both in pair**	3,590,695 / 94.4%	3,590,679 / 94.5%	3,527,381 / 92.77%	3,586,298 / 94.37%	3,406,934 / 94.88%	3,527,381 / 92.77%	3,406,918 / 94.98%	3,406,614 / 94.98%	3,406,614 / 94.98%
**Mapped reads, singletons**	38,058 / 1%	38,058 / 1%	40,732 / 1.07%	37,468 / 0.99%	520 / 0.01%	40,732 / 1.07%	520 / 0.01%	577 / 0.02%	577 / 0.02%
**Read min/max/mean length**	29 / 76 / 75.92	21 / 76 / 75.92	21 / 76 / 71.74	30 / 66 / 65.97	29 / 76 / 75.93	21 / 76 / 71.74	21 / 76 / 75.93	21 / 76 / 74.82	21 / 76 / 74.82
**Clipped reads**	143,865 / 3.78%	143,808 / 3.78%	64,753 / 1.7%	81,541 / 2.15%	93,103 / 2.59%	64,748 / 1.7%	93,030 / 2.59%	68,252 / 1.9%	68,249 / 1.9%
**Duplication rate**	15.08%	15.08%	14.94%	14.70%	14.59%	14.94%	14.59%	14.70%	14.70%
**GC (%)**	39.11%	39.11%	39.00%	39.08%	39.06%	39.00%	39.06%	38.99%	38.99%
**Total reads with indels**	13,683	13,690	8,350	10,925	10,756	8,352	10,761	8,413	8,409
**Insertions**	5,144	5,152	2,855	3,934	3,951	2,853	3,941	2,924	2,921
**Deletions**	8,539	8,538	5,495	6,991	6,805	5,499	6,820	5,489	5,488
**Mean insert size**	169.86	169.87	170.07	169.79	170.12	170.06	170.12	170.18	170.18
**Median insert size**	154	154	154	154	154	154	154	154	154
**Mapping runtime (mins)**	1.32	0.83	0.93	1.01	0.68	0.82	0.83	0.73	0.8

Table SA4-3 Summary of *Escherichia coli O157 H7* reference-based assemblies of raw and trimmed data generated by *ngsShoRT* algorithms

DataSet	Raw	1_5adpt	2_TERA	3_3end	4_LQR	5_Mott	6-LQR-5adpt	7-LQR-5adpt-TERA	8_LQR-5adpt-Mott
**Reference genome size**	5,498,450	5,498,450	5,498,450	5,498,450	5,498,450	5,498,450	5,498,450	5,498,450	5,498,450
**Number of reads**	4,490,536	4,490,534	4,490,265	4,489,637	4,353,255	4,490,265	4,353,253	4,353,151	4,353,151
**Mapped reads**	4,347,142 / 96.81%	4,347,138 / 96.81%	4,335,353 / 96.55%	4,345,328 / 96.79%	4,218,371 / 96.9%	4,335,353 / 96.55%	4,218,368 / 96.9%	4,218,103 / 96.9%	4,218,103 / 96.9%
**Unmapped reads**	143,394 / 3.19%	143,396 / 3.19%	154,912 / 3.45%	144,309 / 3.21%	134,884 / 3.1%	154,912 / 3.45%	134,885 / 3.1%	135,048 / 3.1%	135,048 / 3.1%
**Paired reads**	4,347,142 / 96.81%	4,347,137 / 96.81%	4,335,353 / 96.55%	4,345,328 / 96.79%	4,102,953 / 94.25%	4,335,353 / 96.55%	4,102,949 / 94.25%	4,102,684 / 94.25%	4,102,684 / 94.25%
**Mapped reads, only first in pair**	2,175,571 / 48.45%	2,175,570 / 48.45%	2,174,913 / 48.44%	2,174,613 / 48.44%	2,051,407 / 47.12%	2,174,913 / 48.44%	2,051,406 / 47.12%	2,051,320 / 47.12%	2,051,320 / 47.12%
**Mapped reads, only second in pair**	2,171,571 / 48.36%	2,171,567 / 48.36%	2,160,440 / 48.11%	2,170,715 / 48.35%	2,051,546 / 47.13%	2,160,440 / 48.11%	2,051,543 / 47.13%	2,051,364 / 47.12%	2,051,364 / 47.12%
**Mapped reads, both in pair**	4,340,744 / 96.66%	4,340,737 / 96.66%	4,318,400 / 96.17%	4,338,554 / 96.63%	4,101,058 / 94.21%	4,318,400 / 96.17%	4,101,053 / 94.21%	4,100,698 / 94.2%	4,100,698 / 94.2%
**Mapped reads, singletons**	6,398 / 0.14%	6,400 / 0.14%	16,953 / 0.38%	6,774 / 0.15%	1,895 / 0.04%	16,953 / 0.38%	1,896 / 0.04%	1,986 / 0.05%	1,986 / 0.05%
**Read min/max/mean length**	30 / 150 / 149.85	30 / 150 / 149.44	21 / 150 / 144.04	30 / 140 / 139.88	30 / 150 / 149.85	21 / 150 / 144.04	30 / 150 / 149.43	30 / 150 / 147.01	30 / 150 / 147.01
**Clipped reads**	817,083 / 18.2%	775,935 / 17.28%	665,970 / 14.83%	537,645 / 11.98%	756,534 / 17.38%	665,960 / 14.83%	716,072 / 16.45%	628,986 / 14.45%	628,973 / 14.45%
**Duplication rate**	37.31%	37.36%	37.14%	36.84%	36.60%	37.15%	36.66%	36.62%	36.63%
**GC (%)**	50.86%	50.87%	50.72%	50.86%	50.76%	50.72%	50.76%	50.69%	50.69%
**Total reads with indels**	21,384	21,396	17,697	19,387	19,109	17,701	19,102	17,656	17,661
**Insertions**	9,452	9,459	7,489	8,433	8,357	7,494	8,353	7,475	7,477
**Deletions**	11,932	11,937	10,208	10,954	10,752	10,207	10,749	10,181	10,184
**Mean insert size**	198.75	198.75	198.33	198.16	198.14	198.33	198.15	198.04	198.04
**Median insert size**	188	188	187	188	187	187	187	187	187
**Mapping runtime (mins)**	0.98	0.96	0.88	1.06	0.76	0.83	0.78	0.73	0.75
