# Supplementary material for: Software for pre-processing Illumina next-generation sequencing short read sequences
Source: Source Code Biol Med. 2014 May 3;9:8. doi: 10.1186/1751-0473-9-8 (PMC4064128; doi:10.1186/1751-0473-9-8)
Supplement: Additional file 3: Table SA5-1 — Summary of Caenorhabditis elegans trimmed data generated by ngsShoRT algorithms and other tools. Table SA5-2. Summary of Saccharomyces cerevisiae S288c trimmed data generated by ngsShoRT algorithms and other tools. Table SA5-3. Summary of Escherichia coli O157 H7 trimmed data generated by ngsShoRT algorithms and other tools. Table SA6-1. Summary of Caenorhabditis elegans de novo genome assemblies of raw and trimmed data generated by ngsShoRT algorithms and other tools. Table SA6-2. Summary of Saccharomyces cerevisiae S288c de novo genome assemblies of raw and trimmed data generated by ngsShoRT algorithms and other tools. Table SA6-3. Summary of Escherichia coli O157 H7 de novo genome assemblies of raw and trimmed data generated by ngsShoRT algorithms and other tools. Table SA7-1. Correctness of Caenorhabditis elegans de novo genome assemblies of raw and trimmed data generated by ngsShoRT algorithms and other tools. Table SA7-2. Correctness of Saccharomyces cerevisiae S288c de novo genome assemblies of raw and trimmed data generated by ngsShoRT algorithms and other tools. Table SA7-3. Correctness of Escherichia coli O157 H7 de novo genome assemblies of raw and trimmed data generated by ngsShoRT algorithms and other tools. Table SA8-1. Summary of Caenorhabditis elegans reference-based assemblies of raw and trimmed data generated by ngsShoRT algorithms and other tools. Table SA8-2. Summary of Saccharomyces cerevisiae S288c reference-based assemblies of raw and trimmed data generated by ngsShoRT algorithms and other tools. Table SA8-3. Summary of Escherichia coli O157 H7 reference-based assemblies of raw and trimmed data generated by ngsShoRT algorithms and other tools. [file 1751-0473-9-8-S3.pdf]

Table SA5-1 Summary of *Caenorhabditis elegans* trimmed data generated by *ngsShoRT* algorithms and other tools

**Method category**	**Adapter trimming**				**3' end trimming**		**Quality score based trimming**					
**Tools**	ngsShoRT (v 2.1)	NGS QC Toolkit (v 2.3.2)	Trimmomatic (v 0.32)	cutAdpt (v 1.3)	ngsShoRT (v 2.1)	NGS QC Toolkit (v 2.3.2)	ngsShoRT (v 2.1)	ngsShoRT (v 2.1)	ngsShoRT (v 2.1)	NGS QC Toolkit (v 2.3.2)	NGS QC Toolkit (v 2.3.2)	Trimmomatic (v 0.32)
**DataSet**	1_5adpt	1_5adpt	1_illuminaAdpt	1_cutAdpt	3_3end	2_3end	2_TERA	4_LQR	5_Mott	3_LQR	4_qualCutoff	2_slidingWindow
**# threads**	32	32	32	1	32	1	32	32	32	32	1	32
**Trimming algorithm**	5adpt	IlluQC.pl (adapter trimming)	ILLUMINACLIP	Global removal of adapter sequences	3end	TrimmingReads.pl (3' end trimming)	tera	lqr	mott	IlluQC.pl (Low quality trimming)	TrimmingReads. pl (Quality cutoff trimming)	SLIDINGWINDOW
**Trimming algorithm parameters**	mrl: 21, list=Illumina adapter, action = ka, mp = 100	2 A –p 32	PE -threads 32 -phred33 ILLUMINACLIP:pe _pattern.fa :2:30:12 MINLEN:21	-m 21 –b <adapter sequences>	mrl: 21, x=10	–n 21 –r 5	mrl: 21, avg = 2	mrl: 21, lqs = 4, p = 50	mrl: 21, ml=0.6	N A –l 50 2 -p 32	–n 21 –q 2	PE -threads 32 -phred33 SLIDINGWINDOW:10:2 MINLEN:21
**Mean Phred quality score**	29.53	31.37	29.55	29.58	30.33	29.95	31.57	30.55	31.57	30.64	29.51	29.53
**Low Phred quality score (<=10)**	1,899,444 (2.81%)	0 (0.00%)	1,894,826 (2.80%)	1905212 (2.82%)	1,741,324 (2.58%)	1,815,074 (2.68%)	835,813 (1.24%)	256 (0.00%)	835825 (1.24%)	0 (0.00%)	1,874,272 (2.77%)	1,808,179 (2.68%)
**Total runtime (mins)**	23.51	667.78	41	325.52	14.76	18.9	18.31	18.58	24.65	449.22	35.05	39.5
**Peak thread runtime (mins)**	9.35	NA	NA	NA	6.05	NA	8.05	8.58	15.31	NA	NA	NA
**Merging runtime (mins)**	14.03	NA	NA	NA	8.6	NA	10.18	10.18	9.21	NA	NA	NA
**Peak RAM usage (MB)**	84.5	15.55	11460.72	6.66	81.87	3.95	83.17	83.42	85.12	15.5	3.95	3.96
**Total sequences (paired)**	33,804,923	27,481,664	33,802,324	33,806,467	33,808,546	33,808,546	33,808,546	31,136,730	33,808,546	30,895,102	33,787,215	33,752,156
**Total bases (paired)**	6,727,619,415	5,496,332,800	6,713,730,648	6,645,464,603	6,085,538,280	6,423,623,740	6,198,092,855	6,227,346,000	6,198,004,714	6,179,020,400	6,756,628,181	6,747,516,571
**Total sequences (single)**	2,748	4,510,854	4,634	0	0	0	0	2,103,478	0	2,201,411	0	56,242
**Total bases (single)**	274,511	451,085,400	461,220	0	0	0	0	210,347,800	0	220,141,100	0	4,552,569
**Total bases**	6,727,893,926	5,947,418,200	6,714,191,868	6,645,464,603	6,085,538,280	6,423,623,740	6,198,092,855	6,437,693,800	6,198,004,714	6,399,161,500	6,756,628,181	6,752,069,140

Table SA5-2 Summary of *Saccharomyces cerevisiae S288c* trimmed data generated by *ngsShoRT* algorithms and other tools

**Method category**	**Adapter trimming**				**3' end trimming**		**Quality score based trimming**					
**Tools**	ngsShoRT (v 2.1)	NGS QC Toolkit (v 2.3.2)	Trimmomatic (v 0.32)	cutAdpt (v 1.3)	ngsShoRT (v 2.1)	NGS QC Toolkit (v 2.3.2)	ngsShoRT (v 2.1)	ngsShoRT (v 2.1)	ngsShoRT (v 2.1)	NGS QC Toolkit (v 2.3.2)	NGS QC Toolkit (v 2.3.2)	Trimmomatic (v 0.32)
**DataSet**	1_5adpt	1_5adpt	1_illuminaAdpt	1_cutAdpt	3_3end	2_3end	2_TERA	4_LQR	5_Mott	3_LQR	4_qualCutoff	2_slidingWindow
**# threads**	32	32	32	1	32	1	32	32	32	32	1	32
**Trimming algorithm**	5adpt	IlluQC.pl	ILLUMINACLIP	Global removal of adapter sequences	3end	TrimmingReads.pl	tera	lqr	mott	IlluQC.pl	TrimmingReads. pl (Quality cutoff trimming)	SLIDINGWINDOW
**Trimming algorithm parameters**	mrl: 21, list=Illumina adapter, action = ka, mp = 100	2 A –p 32	PE -threads 32 -phred33 ILLUMINACLIP:pe _pattern.fa:	-m 21 –b <adapter sequences>	mrl: 21, x=10	–n 21 –r 5	mrl: 21, avg = 2	mrl: 21, lqs = 4, p = 50	mrl: 21, ml=0.6	N A –l 50 2 -p 32	–n 21 –q 2	PE -threads 32 -phred33 SLIDINGWINDOW:10:2 MINLEN:21
**Mean Phred quality score**	34.18	36.34	34.22	34.23	34.63	34.43	35.08	35.86	35	35.95	34.17	34.2
**Low Phred quality score (<=10)**	167,587 (4.42%)	0 (0.00%)	165,289 (4.38%)	165,830 (4.39%)	159,108 (4.19%)	163,289 (4.30%)	119,114 (3.14%)	4 (0.00%)	119,114 (3.14%)	0 (0.00%)	167,669 (4.42%)	164,832 (4.34%)
**Total runtime (mins)**	1.45	30.9	2.25	30.3	0.93	1.15	1.01	1.08	1.42	19.52	1.55	2.52
**Peak thread runtime (mins)**	0.7	NA	NA	NA	0.4	NA	0.45	0.53	0.88	NA	NA	NA
**Merging runtime (mins)**	0.7	NA	NA	NA	0.53	NA	0.56	0.55	0.53	NA	NA	NA
**Peak RAM usage (MB)**	82.43	15.33	10237.7266	6.39	81.7	3.95	81.78	84.12	81.91	15.27	3.95	7287.738
**Total sequences (paired)**	1,894,251	1,666,403	1,885,399	1,889,919	1,898,259	1,898,259	1,898,259	1,740,787	1,898,259	1,731,604	1,898,259	1,896,133
**Total bases (paired)**	287,920,526	253,293,256	286,566,814	283,553,600	250,570,188	269,552,778	272,556,467	264,599,624	272,556,067	263,203,808	288,535,368	288,127,853
**Total sequences (single)**	4,008	146,285	5,005	0	0	0	0	102,619	0	104,871	0	1,867
**Total bases (single)**	304,608	11,117,660	380,380	0	0	0	0	7,799,044	0	7,970,196	0	134,767
**Total bases**	288,225,134	264,410,916	286,947,194	283,553,600	250,570,188	269,552,778	272,556,467	272,398,668	272,556,067	271,174,004	288,535,368	288,262,620

Table SA5-3 Summary of *Escherichia coli O157 H7* trimmed data generated by *ngsShoRT* algorithms and other tools

**Method category**	**Adapter trimming**				**3’ end trimming**		**Quality score based trimming**					
**Tools**	ngsShoRT (v 2.1)	NGS QC Toolkit (v 2.3.2)	Trimmomatic (v 0.32)	cutAdpt (v 1.3)	ngsShoRT (v 2.1)	NGS QC Toolkit (v 2.3.2)	ngsShoRT (v 2.1)	ngsShoRT (v 2.1)	ngsShoRT (v 2.1)	NGS QC Toolkit (v 2.3.2)	NGS QC Toolkit (v 2.3.2)	Trimmomatic (v 0.32)
**DataSet**	1_5adpt	1_5adpt	1_illuminaAdpt	1_cutAdpt	3_3end	2_3end	2_TERA	4_LQR	5_Mott	3_LQR	4_qualCutoff	2_slidingWindow
**# threads**	32	32	32	1	32	1	32	32	32	32	1	32
**Trimming algorithm**	5adpt	IlluQC.pl	ILLUMINACLIP	Global removal of adapter sequences	3end	TrimmingReads.pl	tera	lqr	mott	IlluQC.pl	TrimmingReads.pl (Quality cutoff trimming)	SLIDINGWINDOW
**Trimming algorithm parameters**	mrl: 21, list=Illumina adapter, action = ka, mp = 100	2 A –p 32	PE -threads 32 -phred33 ILLUMINACLIP:pe_pattern.fa:2:30:12 MINLEN:21	-m 21 –b <adapter sequences>	mrl: 21, x=10	–n 21 –r 5	mrl: 21, avg = 2	mrl: 21, lqs = 4, p = 50	mrl: 21, ml=0.6	N A –l 50 2 -p 32	–n 21 –q 2	PE -threads 32 -phred33 SLIDINGWINDOW:10:2 MINLEN:21
**Mean Phred quality score**	33.14	34.32	33.16	33.21	33.5	33.12	34.17	33.85	34.17	34.5	33.31	33.16
**Low Phred quality score (<=10)**	76,569 (1.71%)	0 (0.00%)	76,389 (1.70%)	76,291 (1.70%)	69,820 (1.56%)	76,598 (1.71%)	10,492 (0.23%)	0 (0.00%)	10492 (0.23%)	0 (0.0%)	73,257 (1.63%)	69,856 (1.56%)
**Total runtime (mins)**	1.9	58.65	2.5	27.35	1.25	1.67	1.43	1.53	2.25	43.83	3.18	2.62
**Peak thread runtime (mins)**	0.9	NA	NA	NA	0.52	NA	0.67	0.78	1.51	NA	NA	NA
**Merging runtime (mins)**	0.98	NA	NA	NA	0.73	NA	0.76	0.73	0.71	NA	NA	NA
**Peak RAM usage (MB)**	82.09	16.09	10324.88	6.39	83.48	83.44	82.76	82.95	84.98	16.02	3.86	9525.95
**Total sequences (paired)**	2,241,777	1,996,174	2,241,736	2,241,777	2,241,778	2,241,778	2,241,778	2,113,808	2,241,778	2,098,815	2,241,778	2,240,861
**Total bases (paired)**	670,699,844	598,852,200	668,304,902	661,536,106	627,697,840	650,115,620	646,423,163	634,142,400	646,418,288	629,644,500	672,533,400	671,174,112
**Total sequences (single)**	1	209,785	41	0	0	0	0	118,827	0	129,472	0	917
**Total bases (single)**	150	31,467,750	6,088	0	0	0	0	17,824,050	0	19,420,800	0	100,953
**Total bases**	670,699,994	630,319,950	668,310,990	661,536,106	627,697,840	650,115,620	646,423,163	651,966,450	646,418,288	649,065,300	672,533,400	671,275,065

Table SA6-1 Summary of *Caenorhabditis elegans de novo* genome assemblies of raw and trimmed data generated by *ngsShoRT* algorithms and other tools

**Method category**	**Raw**	**Adapter trimming**				**3' end trimming**		**Quality score based trimming**					
**Tools**	NA	ngsShoRT (v 2.1)	NGS QC Toolkit (v 2.3.2)	Trimmomatic (v 0.32)	cutAdpt (v 1.3)	ngsShoRT (v 2.1)	NGS QC Toolkit (v 2.3.2)	ngsShoRT (v 2.1)	ngsShoRT (v 2.1)	ngsShoRT (v 2.1)	NGS QC Toolkit (v 2.3.2)	NGS QC Toolkit (v 2.3.2)	Trimmomatic (v 0.32)
**DataSet**	raw	1_5adpt	1_5adpt	1_illuminaAdpt	1_cutAdpt	3_3end	2_3end	2_TERA	4_LQR	5_Mott	3_LQR	4_qualCutoff	2_slidingWindow
**VelvetOptimiser K-mer range**	21 to 71, step 2	21 to 71, step 2	21 to 71, step 2	21 to 71, step 2	21 to 71, step 2	21 to 71, step 2	21 to 71, step 2	21 to 71, step 2	21 to 71, step 2	21 to 71, step 2	21 to 71, step 2	21 to 71, step 2	21 to 71, step 2
**Optimal K-mer**	59	57	59	53	59	55	53	53	59	53	55	57	57
**Total contigs length (bp)**	99,712,970	100,450,601	100,516,903	100,083,943	100,617,975	100,319,545	100,100,171	100,071,634	100,597,965	100,060,740	100,251,062	100,453,705	100,445,418
**Total # contigs**	10,789	10,404	11,821	10,481	10,522	10,383	10,393	10,776	10,549	10,755	10,539	10,434	10,443
**Max contig length (bp)**	242,599	313,669	198,439	288,459	305,200	246,594	283,307	354,328	313,984	354,102	391,230	313,681	315,260
**E-size (bp)**	45,484	51,429	39,829	49,851	51,336	49,483	49,758	47,096	50,183	47,424	50,016	50,517	51,663
**N25 (bp)**	62,833	67,636	54,706	68,575	69,166	66,635	68,867	62,934	67,555	63,244	66,536	68,297	68,359
**N50 (bp)**	32,064	35,124	28,149	33,826	34,877	34,566	34,105	32,417	34,320	32,509	33,425	34,979	34,786
**N75 (bp)**	14,163	15,640	12,839	15,492	15,638	15,704	15,561	14,589	15,429	14,684	15,228	15,649	15,569
**N90 (bp)**	5,637	6,187	5,101	6,035	6,153	6,093	6,028	5,709	6,059	5,713	6,034	6,177	6,183
**GC content (%)**	35.78	35.88	35.83	35.9	35.86	35.86	35.89	35.8	35.86	35.8	35.88	35.88	35.88
**Velveth runtime (mins)**	13.85	13.76	8.93	11.38	10.75	10.76	12.01	11.23	10.35	11.5	10.18	11.97	12.47
**Velveth peak RAM usage (MB)**	33292.17	41054.26	20001.48	32008.74	29993.23	26148.76	30176.66	16867.36	23361.7	31367.98	26521.99	33672.33	33508.42
**Velvetg runtime (mins)**	34.18	31.91	49.76	32.93	30.45	31.52	31.93	31.67	32.06	33.8	36.93	32.1	53.13
**Velvetg peak RAM usage (MB)**	16227.09	16250.76	13239.32	18484.9	14749.03	15487.39	17919.77	15450.44	15064.87	15305.32	14289.68	14888.89	15070.54
**Velvet runtime (mins)**	48.03	45.67	58.69	44.31	41.2	42.28	43.94	42.9	42.41	45.3	47.11	44.07	65.6
**Velvet peak RAM usage (MB)**	49519.26	57305.02	33240.8	50493.64	44742.26	41636.15	48096.43	32317.8	38426.57	46673.3	40811.67	48561.22	48578.96

Table SA6-2 Summary of *Saccharomyces cerevisiae S288c de novo* genome assemblies of raw and trimmed data generated by *ngsShoRT* algorithms and other tools

**Method category**	**Raw**	**Adapter trimming**				**3' end trimming**		**Quality score based trimming**					
**Tools**	NA	ngsShoRT (v 2.1)	NGS QC Toolkit (v 2.3.2)	Trimmomatic (v 0.32)	cutAdpt (v 1.3)	ngsShoRT (v 2.1)	NGS QC Toolkit (v 2.3.2)	ngsShoRT (v 2.1)	ngsShoRT (v 2.1)	ngsShoRT (v 2.1)	NGS QC Toolkit (v 2.3.2)	NGS QC Toolkit (v 2.3.2)	Trimmomatic (v 0.32)
**DataSet**	raw	1_5adpt	1_5adpt	1_illuminaAdpt	1_cutAdpt	3_3end	2_3end	2_TERA	4_LQR	5_Mott	3_LQR	4_qualCutoff	2_slidingWindow
**VelvetOptimiser K-mer range**	21 to 71, step 2	21 to 71, step 2	21 to 71, step 2	21 to 71, step 2	21 to 71, step 2	21 to 71, step 2	21 to 71, step 2	21 to 71, step 2	21 to 71, step 2	21 to 71, step 2	21 to 71, step 2	21 to 71, step 2	21 to 71, step 2
**Optimal K-mer**	31	31	33	33	31	31	29	33	31	33	31	33	33
**Total contigs length (bp)**	11,459,063	11,458,557	11,468,932	11470657	11457918	11,453,735	11,440,356	11,471,167	11,459,492	11,471,030	11459931	11470835	11470167
**Total # contigs**	1097	1,032	1,124	1035	1040	1,135	1,054	1,057	1,053	1,057	1053	1037	1037
**Max contig length (bp)**	101,217	123,093	102,644	102660	106888	95,726	117,730	102,654	102,663	102,654	102663	102660	102653
**E-size (bp)**	27,917	32,412	28,109	32,113	31,853	28,027	30,761	30,607	31,744	30,610	31,886	31,661	31,853
**N25 (bp)**	39,313	46,832	40,339	47,012	46832	39,910	44,394	45,306	46,227	45,306	46,227	46,188	47,012
**N50 (bp)**	26,221	29,080	25,749	30,443	29062	26,386	28,049	28,539	28,753	28,692	28,782	30,443	30,443
**N75 (bp)**	14,483	16,450	14,312	16,395	16134	13,864	15,461	15,439	15,642	15,439	15,651	16,114	16,395
**N90 (bp)**	7,100	8,320	6,801	8,207	8256	6,866	7,841	7,384	7,828	7,708	7,828	8,207	8,147
**GC content (%)**	38.1	38.07	38.07	38.07	38.07	38.06	38.08	38.06	38.08	38.06	38.08	38.07	38.07
**Velveth runtime (mins)**	0.5	0.47	0.57	0.58	0.57	0.42	0.62	0.42	0.42	0.43	0.67	0.62	0.42
**Velveth peak RAM usage (MB)**	2386.38	1953.91	1,355.75	1,937.99	1,920.93	1701.79	1,831.51	1972.8	1475.32	1972.27	1,453.19	1,943.86	2,512.92
**Velvetg runtime (mins)**	1.58	1.62	1.55	1.65	1.7	1.45	1.73	1.45	1.52	1.5	1.63	1.65	1.65
**Velvetg peak RAM usage (MB)**	1528.89	1541.94	1,368.63	1,454.62	1,473.10	1401.07	1,446.17	1457.18	1474.82	1457.29	1,419.25	1,463	1,459.25
**Velvet runtime (mins)**	2.08	2.09	2.12	2.23	2.27	1.87	2.35	1.87	1.94	1.93	2.30	2.27	2.07
**Velvet peak RAM usage (MB)**	3915.27	3495.85	2,724.38	3,392.61	3,394.03	3,102.86	3,277.68	3,429.98	2,950.14	3,429.56	2,872.44	3,406.86	3,972.17

Table SA6-3 Summary of *Escherichia coli O157 H7 de novo* genome assemblies of raw and trimmed data generated by *ngsShoRT* algorithms and other tools

**Method category**	**Raw**	**Adapter trimming**				**3' end trimming**		**Quality score based trimming**					
**Tools**	NA	ngsShoRT (v 2.1)	NGS QC Toolkit (v 2.3.2)	Trimmomatic (v 0.32)	cutAdpt (v 1.3)	ngsShoRT (v 2.1)	NGS QC Toolkit (v 2.3.2)	ngsShoRT (v 2.1)	ngsShoRT (v 2.1)	ngsShoRT (v 2.1)	NGS QC Toolkit (v 2.3.2)	NGS QC Toolkit (v 2.3.2)	Trimmomatic (v 0.32)
**DataSet**	raw	1_5adpt	1_5adpt	1_illuminaAdpt	1_cutAdpt	3_3end	2_3end	2_TERA	4_LQR	5_Mott	3_LQR	4_qualCutoff	2_slidingWindow
**VelvetOptimiser K-mer range**	21 to 101, step 2	21 to 101, step 2	21 to 101, step 2	21 to 101, step 2	21 to 101, step 2	21 to 101, step 2	21 to 101, step 2	21 to 101, step 2	21 to 101, step 2	21 to 101, step 2	21 to 101, step 2	21 to 101, step 2	21 to 101, step 2
**Optimal K-mer**	65	83	101	83	73	61	71	81	63	53	89	65	83
**Total contigs length (bp)**	5,343,509	5,377,817	5,428,955	5,377,979	5,363,693	5,340,366	5,360,433	5,376,030	5,343,371	5,318,483	5,388,881	5,346,357	5,377,774
**Total # contigs**	417	429	565	432	425	413	416	426	415	413	453	409	431
**Max contig length (bp)**	440,689	440,768	351,081	440,779	440,710	440,609	440,697	440,755	440,676	440,600	351,610	440,690	440,793
**E-size (bp)**	144,862	154,338	143,353	153,376	155,305	1,534,555	157,673	165,801	148206	152,471	149,461	151,010	154,318
**N25 (bp)**	206,077	201,538	215,147	201,556	217,654	222,514	217,650	269,539	217,634	219,582	229,187	217,638	201,556
**N50 (bp)**	138,164	133,303	131,792	133,303	132,682	145,318	145,338	146,563	133,173	145,302	146,666	145,326	132,899
**N75 (bp)**	55,036	58,448	63,079	58,448	58,428	58,398	55,048	65,903	55,026	49,914	63,077	58,406	58,448
**N90 (bp)**	19,524	19,668	15,823	19,668	19,540	21,340	21,352	25,042	21,336	20,376	25,058	19,691	19,668
**GC content (%)**	50.24	50.24	50.27	50.25	50.25	50.24	50.24	50.25	50.24	50.23	50.26	50.24	50.24
**Velveth runtime (mins)**	0.82	0.8	0.8	0.83	0.87	0.83	0.88	0.75	0.83	0.86	0.92	1.11	0.72
**Velveth peak RAM usage (MB)**	3,039.85	2,823.38	1,999.16	2,686.48	2,533.29	2,436.14	2,688.18	2,921.06	2,434.88	2,932.83	2,339.50	3,042.05	3,758.43
**Velvetg runtime (mins)**	1.98	1.75	1.85	1.85	1.93	1.92	1.92	1.72	1.98	2.18	1.76	2.11	1.87
**Velvetg peak RAM usage (MB)**	1,171.93	1,228.18	1,097.10	1,158.04	1,147.50	1,159.11	1,097.08	1,192.27	1,203.38	1,383.91	1,122.10	1,171.16	1,159.04
**Velvet runtime (mins)**	1.98	1.75	1.85	1.85	1.93	1.92	1.92	1.72	1.98	2.18	1.76	2.11	1.87
**Velvet peak RAM usage (MB)**	1,171.93	1,228.18	1,097.10	1,158.04	1,147.50	1,159.11	1,097.08	1,192.27	1,203.38	1,383.91	1,122.10	1,171.16	1,159.04

Table SA7-1 Correctness of *Caenorhabditis elegans de novo* genome assemblies of raw and trimmed data generated by *ngsShoRT* algorithms and other tools

**Method category**	**Raw**	**Adapter trimming**				**3' end trimming**		**Quality score based trimming**					
**Tools**	NA	ngsShoRT (v 2.1)	NGS QC Toolkit (v 2.3.2)	Trimmomatic (v 0.32)	cutAdpt (v 1.3)	ngsShoRT (v 2.1)	NGS QC Toolkit (v 2.3.2)	ngsShoRT (v 2.1)	ngsShoRT (v 2.1)	ngsShoRT (v 2.1)	NGS QC Toolkit (v 2.3.2)	NGS QC Toolkit (v 2.3.2)	Trimmomatic (v 0.32)
**DataSet**	raw	1_5adpt	1_5adpt	1_illuminaAdpt	1_cutAdpt	3_3end	2_3end	2_TERA	4_LQR	5_Mott	3_LQR	4_qualCutoff	2_slidingWindow
**Reference genome size**	100,286,401	100,286,401	100,286,401	100,286,401	100,286,401	100,286,401	100,286,401	100,286,401	100,286,401	100,286,401	100,286,401	100,286,401	100,286,401
**Reference genome coverage**	98,403,841 (98.12%)	98,822,655 (98.54%)	98,814,916 (98.53%)	98,488,217 (98.21%)	98,929,553 (98.65%)	98,669,067 (98.39%)	98,509,256 (98.23%)	98,392,719 (98.11%)	98,939,675 (98.66%)	98,393,760 (98.11%)	98,640,366 (98.36%)	98,842,219 (98.56%)	98,841,872 (98.56%)
**Total contigs**	10,789	10,404	11,821	10,481	10,522	10,352	10,393	10,776	10,549	10,755	10,539	10,434	10,443
**Aligned contigs**	10,569 (97.96%)	10,242 (98.44%)	11,497 (97.26%)	10,332 (98.58%)	10,365 (98.51%)	10,185 (98.39%)	10,234 (98.47%)	10,611 (98.47%)	10,387 (98.46%)	10,590 (98.47%)	10,383 (98.52%)	10,270 (98.43%)	10,283 (98.47%)
**Avg identity (%) (1-1 mapping)**	99.96	99.96	99.95	99.95	99.96	99.95	99.95	99.94	99.96	99.94	99.95	99.95	99.95
**Avg identity (%) (m-m mapping)**	99.85	99.85	99.85	99.85	99.85	99.85	99.85	99.84	99.85	99.84	99.85	99.85	99.85
**TotalSNPs**	9,140	9,868	8,537	10,738	9,088	9,358	10,362	9,860	8,882	9,717	9,558	9,645	9,539

Table SA7-2 Correctness of *Saccharomyces cerevisiae S288c de novo* genome assemblies of raw and trimmed data generated by *ngsShoRT* algorithms and other tools

**Method category**	**Raw**	**Adapter trimming**				**3' end trimming**		**Quality score based trimming**					
**Tools**	NA	ngsShoRT (v 2.1)	NGS QC Toolkit (v 2.3.2)	Trimmomatic (v 0.32)	cutAdpt (v 1.3)	ngsShoRT (v 2.1)	NGS QC Toolkit (v 2.3.2)	ngsShoRT (v 2.1)	ngsShoRT (v 2.1)	ngsShoRT (v 2.1)	NGS QC Toolkit (v 2.3.2)	NGS QC Toolkit (v 2.3.2)	Trimmomatic (v 0.32)
**DataSet**	raw	1_5adpt	1_5adpt	1_illuminaAdpt	1_cutAdpt	3_3end	2_3end	2_TERA	4_LQR	5_Mott	3_LQR	4_qualCutoff	2_slidingWindow
**Reference genome size**	12,157,105	12,157,105	12,157,105	12,157,105	12,157,105	12,157,105	12,157,105	12,157,105	12,157,105	12,157,105	12,157,105	12,157,105	12,157,105
**Reference genome coverage**	11,738,621 (96.56%)	11,761,633 (96.75%)	11,809,050 (97.14%)	11,781,549 (96.91%)	11766257 (96.79%)	11,734,644 (96.52%)	11,731,863 (96.50%)	11,806,630 (97.12%)	11,789,294 (96.97%)	11,807,310 (97.12%)	11,791,068 (96.99%)	11,774,280 (96.85%)	11,775,741 (96.86%)
**Total contigs**	1,097	1,032	1,124	1,035	1,040	1,135	1,054	1,057	1,053	1,057	1,053	1,037	1,037
**Aligned contigs**	1,090 (99.36%)	1,018 (98.64%)	1,116 (99.29%)	1.023 (98.84%)	1.027 (98.75%)	1,114 (98.15%)	1,039 (98.58%)	1,050 (99.34%)	1,041 (98.86%)	1,050 (99.34%)	1,041 (98.86%)	1,025 (98.84%)	1,025 (98.84%)
**Avg identity (%) (1-1 mapping)**	99.96	99.97	99.97	99.97	99.97	99.95	99.97	99.96	99.97	99.96	99.98	99.97	99.97
**Avg identity (%) (m-m mapping)**	99.95	99.95	99.95	99.95	99.95	99.94	99.95	99.94	99.96	99.94	99.96	99.95	99.95
**TotalSNPs**	786	730	621	729	739	781	882	598	654	602	635	713	716

Table SA7-3 Correctness of *Escherichia coli O157 H7 de novo* genome assemblies of raw and trimmed data generated by *ngsShoRT* algorithms and other tools

**Method category**	**Raw**	**Adapter trimming**				**3' end trimming**		**Quality score based trimming**					
**Tools**	NA	ngsShoRT (v 2.1)	NGS QC Toolkit (v 2.3.2)	Trimmomatic (v 0.32)	cutAdpt (v 1.3)	ngsShoRT (v 2.1)	NGS QC Toolkit (v 2.3.2)	ngsShoRT (v 2.1)	ngsShoRT (v 2.1)	ngsShoRT (v 2.1)	NGS QC Toolkit (v 2.3.2)	NGS QC Toolkit (v 2.3.2)	Trimmomatic (v 0.32)
**DataSet**	raw	1_5adpt	1_5adpt	1_illuminaAdpt	1_cutAdpt	3_3end	2_3end	2_TERA	4_LQR	5_Mott	3_LQR	4_qualCutoff	2_slidingWindow
**Reference genome size**	5,498,450	5,498,450	5,498,450	5,498,450	5,498,450	5,498,450	5,498,450	5,498,450	5,498,450	5,498,450	5,498,450	5,498,450	5,498,450
**Reference genome coverage**	5363955 (97.55%)	5384193 (97.92%)	5,389,948 (98.03%)	5,384,971 (97.94%)	5,378,185 (97.81%)	5361630 (97.51%)	5,376,324 (97.78%)	5383225 (97.90%)	5366345 (97.60%)	5336300 (97.05%)	5,385,756 (97.95%)	5,365,800 (97.59%)	5,384,090 (97.92%)
**Total contigs**	417	429	565	432	425	413	416	426	415	413	453	409	431
**Aligned contigs**	409 (98.08%)	427 (99.53%)	563 (99.65%)	430 (99.54%)	420 (98.82%)	406 (98.31%)	411 (98.80%)	423 (99.30%)	407 (98.07%)	401 (97.09%)	449 (99.12%)	401 (98.04%)	429 (99.54%)
**Avg identity (%) (1-1 mapping)**	99.95	99.95	99.96	99.95	99.95	99.95	99.96	99.95	99.96	99.95	99.95	99.95	99.95
**Avg identity (%) (m-m mapping)**	99.9	99.89	99.9	99.89	99.89	99.9	99.9	99.9	99.9	99.91	99.9	99.89	99.9
**TotalSNPs**	1,859	1,929	1,758	1,936	1,952	1,823	1,766	1,899	1,847	1,826	1,853	1,815	1912

Table SA8-1 Summary of *Caenorhabditis elegans* reference-based assemblies of raw and trimmed data generated by *ngsShoRT* algorithms and other tools

**Method category**	**Raw**	**Adapter trimming**				**3' end trimming**		**Quality score based trimming**					
**Tools**	NA	ngsShoRT (v 2.1)	NGS QC Toolkit (v 2.3.2)	Trimmomatic (v 0.32)	cutAdpt (v 1.3)	ngsShoRT (v 2.1)	NGS QC Toolkit (v 2.3.2)	ngsShoRT (v 2.1)	ngsShoRT (v 2.1)	ngsShoRT (v 2.1)	NGS QC Toolkit (v 2.3.2)	NGS QC Toolkit (v 2.3.2)	Trimmomatic (v 0.32)
**DataSet**	raw	1_5adpt	1_5adpt	1_illuminaAdpt	1_cutAdpt	3_3end	2_3end	2_TERA	4_LQR	5_Mott	3_LQR	4_qualCutoff	2_slidingWindow
**Reference genome size**	100,286,401	100,286,401	100,286,401	100,286,401	100,286,401	100,286,401	100,286,401	100,286,401	100,286,401	100,286,401	100,286,401	100,286,401	100,286,401
**Number of reads**	68,029,090	68,024,577	59,825,255	68,016,822	68,016,262	67,901,888	67,962,815	67,948,294	64,751,598	67,948,294	64,363,972	67,986,333	68,003,303
**Mapped reads**	65,108,539 / 95.71%	65,107,835 / 95.71%	57,910,084 / 96.8%	65,107,158 / 95.72%	65,098,109 / 95.71%	64,966,762 / 95.68%	65,035,027 / 95.69%	64,580,965 / 95.04%	62,368,674 / 96.32%	64,582,097 / 95.05%	62,006,146 / 96.34%	65,087,939 / 95.74%	65,101,496 / 95.73%
**Unmapped reads**	2,920,551 / 4.29%	2,916,742 / 4.29%	1,915,171 / 3.2%	2,909,664 / 4.28%	2,918,153 / 4.29%	2,935,126 / 4.32%	2,927,788 / 4.31%	3,367,329 / 4.96%	2,382,924 / 3.68%	3,366,197 / 4.95%	2,357,826 / 3.66%	2,898,394 / 4.26%	2,901,807 / 4.27%
**Paired reads**	65,108,539 / 95.71%	65,107,653 / 95.71%	53,711,670 / 89.78%	65,106,978 / 95.72%	65,098,109 / 95.71%	64,966,762 / 95.68%	65,035,027 / 95.69%	64,580,965 / 95.04%	60,429,886 / 93.33%	64,582,097 / 95.05%	59,971,303 / 93.18%	65,087,939 / 95.74%	65,038,620 / 95.64%
**Mapped reads, only first in pair**	32,603,951 / 47.93%	32,603,560 / 47.93%	26,850,278 / 44.88%	32,603,308 / 47.93%	32,599,886 / 47.93%	32,535,473 / 47.92%	32,568,229 / 47.92%	32,412,953 / 47.7%	30,206,558 / 46.65%	32,414,026 / 47.7%	29,977,483 / 46.57%	32,583,640 / 47.93%	32,555,956 / 47.87%
**Mapped reads, only second in pair**	32,504,588 / 47.78%	32,504,093 / 47.78%	26,861,392 / 44.9%	32,503,670 / 47.79%	32,498,223 / 47.78%	32,431,289 / 47.76%	32,466,798 / 47.77%	32,168,012 / 47.34%	30,223,328 / 46.68%	32,168,071 / 47.34%	29,993,820 / 46.6%	32,504,299 / 47.81%	32,482,664 / 47.77%
**Mapped reads, both in pair**	64,782,848 / 95.23%	64,782,175 / 95.23%	53,615,708 / 89.62%	64,781,797 / 95.24%	64,769,939 / 95.23%	64,631,660 / 95.18%	64,704,768 / 95.21%	64,044,875 / 94.26%	60,307,426 / 93.14%	64,045,411 / 94.26%	59,850,314 / 92.99%	64,782,266 / 95.29%	64,737,254 / 95.2%
**Mapped reads, singletons**	325,691 / 0.48%	325,478 / 0.48%	95,962 / 0.16%	325,181 / 0.48%	328,170 / 0.48%	335,102 / 0.49%	330,259 / 0.49%	536,090 / 0.79%	122,460 / 0.19%	536,686 / 0.79%	120,989 / 0.19%	305,673 / 0.45%	301,366 / 0.44%
**Read min/max/mean length**	30 / 100 / 99.64	21 / 100 / 99.15	30 / 100 / 99.65	21 / 100 / 98.95	21 / 100 / 97.94	30 / 90 / 89.78	30 / 95 / 94.71	21 / 100 / 91.41	30 / 100 / 99.65	21 / 100 / 91.41	30 / 100 / 99.65	21 / 100 / 99.62	21 / 100 / 99.56
**Clipped reads**	6,368,951 / 9.36%	5,746,286 / 8.45%	3,533,838 / 5.91%	5,327,811 / 7.83%	4,638,290 / 6.82%	4,881,239 / 7.19%	5,540,026 / 8.15%	3,555,657 / 5.23%	5,057,053 / 7.81%	3,552,831 / 5.23%	4,983,996 / 7.74%	6,361,176 / 9.36%	6,326,905 / 9.3%
**Duplication rate**	31.14%	31.22%	28.72%	31.30%	34.78%	31.01%	31.07%	30.99%	30.33%	30.99%	30.19%	31.13%	31.16%
**GC (%)**	34.15%	34.15%	33.63%	34.15%	34.17%	34.09%	34.12%	33.74%	33.98%	33.74%	33.97%	34.15%	34.15%
**Total reads with indels**	521,946	522,212	339,285	522,238	510,378	433,970	476,879	330,934	431,268	330,864	425,855	521,979	521,560
**Insertions**	211,101	211,200	132,628	211,187	205,945	175,048	192,453	129,093	171,387	128,999	169,102	211,039	210,921
**Deletions**	310,845	311,012	206,657	311,051	304,433	258,922	284,426	201,841	259,881	201,865	256,753	310,940	310,639
**Mean insert size**	244.42	244.27	244.69	244.27	243.33	244.14	244.2	243.48	243.82	243.47	243.8	244.26	244.26
**Median insert size**	252	252	251	252	251	252	252	252	251	252	251	252	252
**Mapping runtime (mins)**	16.35	16.72	10.3	14.26	18.48	15.88	14.05	15.8	14.28	15.05	16.18	16.28	15.68

Table SA8-2 Summary of *Saccharomyces cerevisiae S288c* reference-based assemblies of raw and trimmed data generated by *ngsShoRT* algorithms and other tools

**Method category**	**Raw**	**Adapter trimming**				**3' end trimming**		**Quality score based trimming**					
**Tools**	NA	ngsShoRT (v 2.1)	NGS QC Toolkit (v 2.3.2)	Trimmomatic (v 0.32)	cutAdpt (v 1.3)	ngsShoRT (v 2.1)	NGS QC Toolkit (v 2.3.2)	ngsShoRT (v 2.1)	ngsShoRT (v 2.1)	ngsShoRT (v 2.1)	NGS QC Toolkit (v 2.3.2)	NGS QC Toolkit (v 2.3.2)	Trimmomatic (v 0.32)
**DataSet**	raw	1_5adpt	1_5adpt	1_illuminaAdpt	1_cutAdpt	3_3end	2_3end	2_TERA	4_LQR	5_Mott	3_LQR	4_qualCutoff	2_slidingWindow
**Reference genome size**	12,157,105	12,157,105	12,157,105	12,157,105	12,157,105	12,157,105	12,157,105	12,157,105	12,157,105	12,157,105	12,157,105	12,157,105	12,157,105
**Number of reads**	3,803,781	3,799,773	3,485,029	3,779,102	3,786,809	3,800,411	3,802,064	3,802,165	3,590,603	3,802,165	3,574,433	3,803,781	3,800,270
**Mapped reads**	3,628,753 / 95.4%	3,628,737 / 95.5%	3,407,828 / 97.78%	3,628,584 / 96.02%	3,627,907 / 95.8%	3,623,766 / 95.35%	3,626,324 / 95.38%	3,568,113 / 93.84%	3,498,316 / 97.43%	3,568,113 / 93.84%	3,484,370 / 97.48%	3,628,753 / 95.4%	3,625,582 / 95.4%
**Unmapped reads**	175,028 / 4.6%	171,036 / 4.5%	77,201 / 2.22%	150,518 / 3.98%	158,902 / 4.2%	176,645 / 4.65%	175,740 / 4.62%	234,052 / 6.16%	92,287 / 2.57%	234,052 / 6.16%	90,063 / 2.52%	175,028 / 4.6%	174,688 / 4.6%
**Paired reads**	3,628,753 / 95.4%	3,628,737 / 95.5%	3,276,439 / 94.01%	3,628,570 / 96.02%	3,627,907 / 95.8%	3,623,766 / 95.35%	3,626,324 / 95.38%	3,568,113 / 93.84%	3,407,454 / 94.9%	3,568,113 / 93.84%	3,391,062 / 94.87%	3,628,753 / 95.4%	3,625,166 / 95.39%
**Mapped reads, only first in pair**	1,805,405 / 47.46%	1,805,397 / 47.51%	1,637,416 / 46.98%	1,805,373 / 47.77%	1,805,133 / 47.67%	1,804,231 / 47.47%	1,804,828 / 47.47%	1,778,833 / 46.78%	1,702,837 / 47.42%	1,778,833 / 46.78%	1,694,656 / 47.41%	1,805,405 / 47.46%	1,803,623 / 47.46%
**Mapped reads, only second in pair**	1,823,348 / 47.94%	1,823,340 / 47.99%	1,639,023 / 47.03%	1,823,197 / 48.24%	1,822,774 / 48.13%	1,819,535 / 47.88%	1,821,496 / 47.91%	1,789,280 / 47.06%	1,704,617 / 47.47%	1,789,280 / 47.06%	1,696,406 / 47.46%	1,823,348 / 47.94%	1,821,543 / 47.93%
**Mapped reads, both in pair**	3,590,695 / 94.4%	3,590,679 / 94.5%	3,276,111 / 94.01%	3,590,643 / 95.01%	3,589,861 / 94.8%	3,586,298 / 94.37%	3,588,624 / 94.39%	3,527,381 / 92.77%	3,406,934 / 94.88%	3,527,381 / 92.77%	3,390,614 / 94.86%	3,590,695 / 94.4%	3,587,326 / 94.4%
**Mapped reads, singletons**	38,058 / 1%	38,058 / 1%	328 / 0.01%	37,927 / 1%	38,046 / 1%	37,468 / 0.99%	37,700 / 0.99%	40,732 / 1.07%	520 / 0.01%	40,732 / 1.07%	448 / 0.01%	38,058 / 1%	37,840 / 1%
**Read min/max/mean length**	29 / 76 / 75.92	21 / 76 / 75.92	29 / 76 / 75.93	21 / 76 / 75.92	21 / 76 / 74.94	30 / 66 / 65.97	30 / 71 / 70.95	21 / 76 / 71.74	29 / 76 / 75.93	21 / 76 / 71.74	29 / 76 / 75.93	29 / 76 / 75.92	21 / 76 / 75.9
**Clipped reads**	143,865 / 3.78%	143,808 / 3.78%	76,646 / 2.2%	143,473 / 3.8%	109,205 / 2.88%	81,541 / 2.15%	104,632 / 2.75%	64,753 / 1.7%	93,103 / 2.59%	64,748 / 1.7%	91,018 / 2.55%	143,865 / 3.78%	142,320 / 3.74%
**Duplication rate**	15.08%	15.08%	14.31%	15.07%	17.89%	14.70%	14.84%	14.94%	14.59%	14.94%	14.54%	15.08%	15.07%
**GC (%)**	39.11%	39.11%	38.98%	39.11%	39.16%	39.08%	39.10%	39.00%	39.06%	39.00%	39.05%	39.11%	39.11%
**Total reads with indels**	13,683	13,690	8,474	13,678	13,081	10,925	12,272	8,350	10,756	8,352	10,534	13,683	13,654
**Insertions**	5,144	5,152	3,028	5,145	4,892	3,934	4,611	2,855	3,951	2,853	3,858	5,144	5,131
**Deletions**	8,539	8,538	5,446	8,533	8,189	6,991	7,661	5,495	6,805	5,499	6,676	8,539	8,523
**Mean insert size**	169.86	169.87	170.35	169.85	168.97	169.79	169.79	170.07	170.12	170.06	170.15	169.86	169.84
**Median insert size**	154	154	155	154	153	154	154	154	154	154	154	154	154
**Mapping runtime (mins)**	1.32	0.83	0.7	0.67	0.62	1.01	0.57	0.93	0.68	1.36	0.52	0.87	0.72

Table SA8-3 Summary of *Escherichia coli O157 H7* reference-based assemblies of raw and trimmed data generated by *ngsShoRT* algorithms and other tools

**Method category**	**Raw**	**Adapter trimming**				**3' end trimming**		**Quality score based trimming**					
**Tools**	NA	ngsShoRT (v 2.1)	NGS QC Toolkit (v 2.3.2)	Trimmomatic (v 0.32)	cutAdpt (v 1.3)	ngsShoRT (v 2.1)	NGS QC Toolkit (v 2.3.2)	ngsShoRT (v 2.1)	ngsShoRT (v 2.1)	ngsShoRT (v 2.1)	NGS QC Toolkit (v 2.3.2)	NGS QC Toolkit (v 2.3.2)	Trimmomatic (v 0.32)
**DataSet**	raw	1_5adpt	1_5adpt	1_illuminaAdpt	1_cutAdpt	3_3end	2_3end	2_TERA	4_LQR	5_Mott	3_LQR	4_qualCutoff	2_slidingWindow
**Reference genome size**	5,498,450	5,498,450	5,498,450	5,498,450	5,498,450	5,498,450	5,498,450	5,498,450	5,498,450	5,498,450	5,498,450	5,498,450	5,498,450
**Number of reads**	4,490,536	4,490,534	4,208,770	4,490,455	4,490,459	4,489,637	4,490,080	4,490,265	4,353,255	4,490,265	4,333,887	4,490,536	4,490,521
**Mapped reads**	4,347,142 / 96.81%	4,347,138 / 96.81%	4,078,188 / 96.9%	4,347,112 / 96.81%	4,346,940 / 96.8%	4,345,328 / 96.79%	4,346,237 / 96.8%	4,335,353 / 96.55%	4,218,371 / 96.9%	4,335,353 / 96.55%	4,199,755 / 96.91%	4,347,142 / 96.81%	4,346,782 / 96.8%
**Unmapped reads**	143,394 / 3.19%	143,396 / 3.19%	130,582 / 3.1%	143,343 / 3.19%	143,519 / 3.2%	144,309 / 3.21%	143,843 / 3.2%	154,912 / 3.45%	134,884 / 3.1%	154,912 / 3.45%	134,132 / 3.09%	143,394 / 3.19%	143,739 / 3.2%
**Paired reads**	4,347,142 / 96.81%	4,347,137 / 96.81%	3,874,143 / 92.05%	4,347,102 / 96.81%	4,346,940 / 96.8%	4,345,328 / 96.79%	4,346,237 / 96.8%	4,335,353 / 96.55%	4,102,953 / 94.25%	4,335,353 / 96.55%	4,073,930 / 94%	4,347,142 / 96.81%	4,345,050 / 96.76%
**Mapped reads, only first in pair**	2,175,571 / 48.45%	2,175,570 / 48.45%	1,936,999 / 46.02%	2,175,553 / 48.45%	2,175,484 / 48.45%	2,174,613 / 48.44%	2,175,081 / 48.44%	2,174,913 / 48.44%	2,051,407 / 47.12%	2,174,913 / 48.44%	2,036,872 / 47%	2,175,571 / 48.45%	2,174,682 / 48.43%
**Mapped reads, only second in pair**	2,171,571 / 48.36%	2,171,567 / 48.36%	1,937,144 / 46.03%	2,171,549 / 48.36%	2,171,456 / 48.36%	2,170,715 / 48.35%	2,171,156 / 48.35%	2,160,440 / 48.11%	2,051,546 / 47.13%	2,160,440 / 48.11%	2,037,058 / 47%	2,171,571 / 48.36%	2,170,368 / 48.33%
**Mapped reads, both in pair**	4,340,744 / 96.66%	4,340,737 / 96.66%	3,872,430 / 92.01%	4,340,708 / 96.67%	4,340,474 / 96.66%	4,338,554 / 96.63%	4,339,658 / 96.65%	4,318,400 / 96.17%	4,101,058 / 94.21%	4,318,400 / 96.17%	4,072,120 / 93.96%	4,340,744 / 96.66%	4,338,361 / 96.61%
**Mapped reads, singletons**	6,398 / 0.14%	6,400 / 0.14%	1,713 / 0.04%	6,394 / 0.14%	6,466 / 0.14%	6,774 / 0.15%	6,579 / 0.15%	16,953 / 0.38%	1,895 / 0.04%	16,953 / 0.38%	1,810 / 0.04%	6,398 / 0.14%	6,689 / 0.15%
**Read min/max/mean length**	30 / 150 / 149.85	30 / 150 / 149.44	30 / 150 / 149.84	21 / 150 / 148.91	30 / 150 / 147.4	30 / 140 / 139.88	30 / 145 / 144.86	21 / 150 / 144.04	30 / 150 / 149.85	21 / 150 / 144.04	30 / 150 / 149.85	30 / 150 / 149.85	21 / 150 / 149.59
**Clipped reads**	817,083 / 18.2%	775,935 / 17.28%	706,065 / 16.78%	694,549 / 15.47%	345,784 / 7.7%	537,645 / 11.98%	670,780 / 14.94%	665,970 / 14.83%	756,534 / 17.38%	665,960 / 14.83%	750,421 / 17.32%	817,083 / 18.2%	808,999 / 18.02%
**Duplication rate**	37.31%	37.36%	35.79%	37.47%	40.13%	36.84%	37.04%	37.14%	36.60%	37.15%	36.49%	37.31%	37.32%
**GC (%)**	50.86%	50.87%	50.65%	50.87%	50.91%	50.86%	50.86%	50.72%	50.76%	50.72%	50.75%	50.86%	50.86%
**Total reads with indels**	21,384	21,396	17,769	21,416	19,792	19,387	20,426	17,697	19,109	17,701	18,952	21,384	21,362
**Insertions**	9,452	9,459	7,731	9,464	8,409	8,433	8,976	7,489	8,357	7,494	8,280	9,452	9,447
**Deletions**	11,932	11,937	10,038	11,952	11,383	10,954	11,450	10,208	10,752	10,207	10,672	11,932	11,915
**Mean insert size**	198.75	198.75	198.01	198.75	197.96	198.16	198.44	198.33	198.14	198.33	198.1	198.75	198.74
**Median insert size**	188	188	187	188	187	188	188	187	187	187	187	188	188
**Mapping runtime (mins)**	0.98	0.96	0.78	0.83	0.82	1.06	0.68	0.88	0.76	0.83	0.62	0.78	0.98
